# The viral BCL2 protein BHRF1 of Epstein–Barr virus promotes AIM2 inflammasome activation to facilitate lytic replication

**DOI:** 10.1371/journal.ppat.1013509

**Published:** 2025-09-22

**Authors:** Qingping Lan, Xiaolin Zhang, Yifan Sun, Jing Yang, Xiaojuan Li, Ersheng Kuang

**Affiliations:** 1 Institute of Human Virology, Zhongshan School of Medicine, Sun Yat-Sen University, Guangzhou, China; 2 Key Laboratory of Tropical Disease Control (Sun Yat-Sen University), Ministry of Education, Guangzhou, China; 3 College of Clinical Medicine, Hubei University of Chinese Medicine, Wuhan, Hubei, China; 4 Hubei Shizhen Laboratory, Wuhan, Hubei, China; University of Wisconsin-Madison, UNITED STATES OF AMERICA

## Abstract

The absent in melanoma 2 (AIM2) protein recognizes viral and naked dsDNA and recruit apoptosis-associated speck-like protein containing CARD (ASC) to initiate inflammasome activation; however, the subversion of AIM2 activation by Epstein–Barr virus (EBV) infection remains unknown. Here, we reveal that the EBV-encoded viral BCL2 protein BHRF1 promotes AIM2 inflammasome activation. The BHRF1 C-terminal domain binds to AIM2 HIN domain and directly promotes dsDNA recognition and AIM2-ASC interaction, consequently cooperates with viral dsDNA to enable inflammasome activation. The single-site mutations R162A and F164A in BHRF1 and E186A in AIM2 abolish their interaction and AIM2 inflammasome activation. BHRF1 recruits AIM2 inflammasome to the mitochondrial compartment and facilitates EBV lytic replication through KAP1 and GSDMD cleavage. BHRF1 deficiency strongly decreases AIM2 inflammasome activation and EBV lytic replication, and reintroduction of wild-type BHRF1 but not the BHRF1 R162A or F164A mutant restores these functions. These results suggest that BHRF1 protein directly promotes the AIM2 inflammasome activation in the mitochondrial compartment to facilitate lytic replication.

## Author summary

The cytosolic sensor AIM2 recognizes viral dsDNA to induce inflammasome activation and is subverted during DNA virus infection; however, whether AIM2 is directly activated by viral proteins remains unknown. Here, we revealed that the viral BCL2 protein BHRF1 of Epstein–Barr virus directly binds to AIM2 and disrupts the autoinhibition of AIM2 to promote the assembly and activation of AIM2 inflammasome in the mitochondrial compartment and then facilitate lytic replication. Our findings revealed that BHRF1 plays an essential role in AIM2 inflammasome activation and facilitates EBV lytic replication through inflammasome activation.

## Introduction

Upon viral infection, pattern recognition receptors (PRRs) recognize viral products and trigger antiviral immune and inflammatory responses [1]. Five types of PRRs have been characterized, including retinoic acid-inducible gene-I (RIG-I)-like receptors (RLRs) and absent in melanoma-2 (AIM2)-like receptors (ALRs), which sense viral RNA and dsDNA [2,3], respectively. The inflammasome is a large innate immune complex that consists of a sensor protein, the adaptor apoptosis-associated speck-like protein containing a caspase recruitment domain (ASC) and the effector pro-caspase-1 to induce Caspase-1 activation and the subsequent cleavage of pro-IL-1β and pro-IL-18 [4,5]. RIG-I and AIM2 mainly recognize viral RNA and dsDNA, respectively, in the cytoplasm to initiate the assembly and activation of inflammasomes [6,7].

AIM2 consists of two main domains, the N-terminal pyrin domain (PYD) and the C-terminal domain of hematopoietic interferon-inducible nuclear proteins with a 200-amino acid motif (HIN) [6]. In the absence of dsDNA binding, AIM2 is inactive via autoinhibition through intramolecular PYD-HIN interactions. Through dsDNA recognition and binding to the HIN domain, the AIM2 PYD domain interacts with the ASC PYD domain, and AIM2 switches from an autoinhibitory state to an active state and recruits ASC and pro-caspase-1 to form a filament along the dsDNA chain [8,9]. Consequently, Caspase-1 is activated by autocleavage and subsequently cleaves several substrates such as IL-1β, IL-18, GSDMD and KAP1, to mediate inflammatory responses, pyroptosis and transcriptional repression/activation. In addition, active AIM2 can recruit other components, such as caspase-8, and form noncanonical inflammasome to trigger apoptosis and PANoptosis [10].

Herpesviruses are a family of dsDNA viruses with large DNA genomes that encode dozens of viral proteins and noncoding RNAs. These viruses establish two unique infections in different cells or tissues: latent infection, which occurs when the virus is dormant within a cell with a few viral latent gene expression and no virion production, and lytic infection, in which the infection is active with whole viral gene expression and the production of progeny virion particles [11]. Eight types of herpesviruses infect humans and cause many diseases, and two types of human oncogenic herpesviruses, Epstein–Barr virus (EBV) and Kaposi’s sarcoma-associated herpesvirus (KSHV), are etiological agents of several malignant cancers and other diseases in humans [12,13].

The infection and replication of herpesviruses results in the production of many viral products, including viral RNA, DNA and proteins, that can be recognized by diverse PRRs. RIG-I and IFI16 are primarily responsible for the recognition of viral RNA and DNA during both the latent and lytic phases of the viral life cycle to activate antiviral innate immune responses and inflammasomes [14–17], and these signaling pathways are also suppressed through different mechanisms during the lytic phase [18–21]. As a prominent cytosolic DNA sensor of the inflammasome, AIM2-mediated inflammasome activation is suppressed by HSV-1 VP22 and KSHV SOX during the lytic life cycle to prevent the antiviral effects of hyperactivation of AIM2 inflammasome and promote lytic replication [22,23]. A low level of inflammasome activation is important for EBV lytic replication [24]; however, the mechanism and function of this process largely remain unknown.

Human oncogenic herpesviruses encode viral BCL2 homologs, including EBV BHRF1 and KSHV vBcl2 (encoded by ORF16), which mainly localize to the outer mitochondrial membrane to suppress mitochondrial dysfunction and apoptosis [25–27] and promote viral lytic replication [28,29]. In addition, BHRF1 stimulates autophagy and inhibits innate immune responses [30], whereas vBcl2 inhibits autophagy [31]. Here, we reveal a new function of EBV BHRF1 in inflammasome activation. BHRF1 interacts with AIM2 and promotes DNA recognition, ASC recruitment and the formation of AIM2 inflammasome in the mitochondrial compartment. Consequently, AIM2 inflammasome activation facilitates EBV lytic replication though KAP1 and GSDMD cleavage without inducing obvious mitochondrial dysfunction. Our findings reveal that BHRF1 promotes AIM2 inflammasome activation and plays important roles in the lytic replication of Epstein–Barr virus.

## Results

### BHRF1 activates the inflammasome during EBV lytic lifecycle

**Fig 1:**
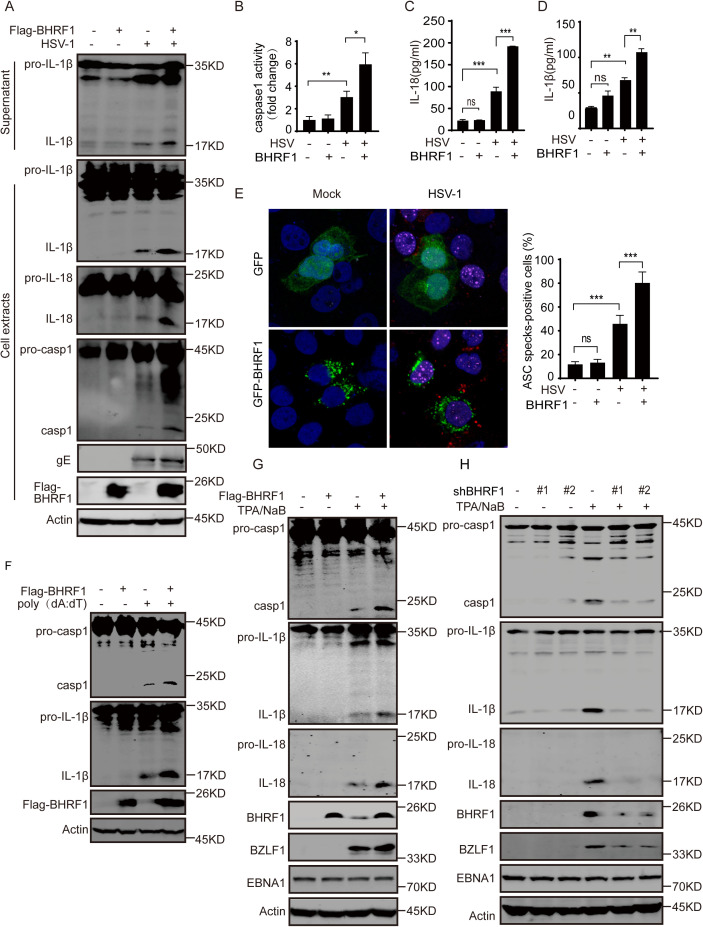
BHRF1 induces inflammasome activation during the EBV lytic lifecycle. A-B. THP-1 cells were infected with empty or BHRF1-expressing lentiviruses for 24 h, primed with 40 ng/ml TPA overnight, and then left uninfected or infected with HSV-1 (MOI = 1) in serum-free medium for 12 h. The supernatants and whole-cell lysates were analyzed by western blotting analysis (A), and the whole-cell lysates were prepared and the relative levels of caspase1 activity were measured and shown (B). Tukey’s multiple comparison test. *, p < 0.5; **, p < 0.05. C-D. The release of IL-1β (B) and IL-18 (C) from THP-1 cells, as described above, was measured by enzyme-linked immunosorbent assay (ELISA). The results are shown as the mean ± SD (n = 3), Tukey’s multiple comparison test. **, p < 0.05; ***, p < 0.005. E. A549 cells were transfected with empty or GFP-BHRF1-expressing plasmids for 36 h, and then left uninfected or infected with HSV-1 (MOI = 1) for 12 h. The cells were fixed and stained with an anti-ASC (red) and anti-ICP0 (purple) antibody. Cells with at least one typical speck were considered as positive, and the expression of ICP0 was detected as evidence of HSV-1 infection. Images of ASC specks were recorded with a fluorescence microscope, three random fields containing about 20-30 cells in each condition were analyzed automatically by ImageJ software, and the percentage of cells with ASC specks was calculated and is shown. The results are shown as the mean ± SD (n = 3), Tukey’s multiple comparison test. ***, p < 0.005. Scale bar: 10 μm. F. THP-1 cells were infected with empty or BHRF1-expressing lentiviruses for 24 h, primed with 40 ng/ml TPA overnight, and then either left untreated or treated with 5 μg/mL poly(dA:dT) for 16 h, whole-cell lysates were prepared and analyzed by western blotting analysis as indicated. G. P3HR1 cells were infected with empty or BHRF1-expressing lentiviruses for 24 h, and then left untreated or treated with TPA plus NaB for 48 h. The cells were collected, and the cell extracts were subjected to western blotting analysis. H. P3HR1 cells were transduced with scramble shRNA or two different shRNAs targeting BHRF1 for 24 h and then left untreated or induced with TPA plus NaB for 48 h. The cell lysates were analyzed by western blotting as indicated.

Our previous study revealed that BRLF1 suppresses RIG-I inflammasome activation and antiviral innate immune responses during EBV lytic replication [19]; however, the increased inflammasome activation and IL-1β secretion are clearly observed at the stage of chemical-induced EBV lytic replication (S1A Fig), whereas this type of treatment rarely induces inflammasome activation in EBV-negative cells (S1B Fig), indicating that there are unknown viral activators of the inflammasome during EBV lytic replication. By screening the EBV ORF-expressing library, we determined that the EBV-encoded BHRF1 induced inflammasome activation (S1C Fig). To investigate the role of BHRF1 in inflammasome activation, THP-1 cells were transiently infected with control or BHRF1-expressing lentiviruses and then infected with HSV-1, which strongly induced inflammasome activation at 12–48 h post infection (S1D Fig). BHRF1 overexpression did not increase inflammasome activation in unstimulated cells but strongly enhanced the cleavage of Caspase-1, IL-1β and IL-18 under HSV-1 infection (Fig 1A), and the activity of Caspase-1 also increased in cells with BHRF1 overexpression compared with the control cells under HSV-1 infection (Fig 1B).The secretion of IL-1β and IL-18 was also greatly increased by BHRF1 overexpression during HSV-1 infection but was only slightly altered under unstimulated conditions (Fig 1C-1D). When ASC specks were counted in the absence or presence of BHRF1 overexpression, BHRF1 minimally affected the formation of ASC specks in control cells but significantly increased it in HSV-1 infected cells (Figs 1E and S1E). Similarly, BHRF1 overexpression enhanced the cleavage of Caspase-1 and IL-1β in cells under poly(dA:dT) stimulation but not in unstimulated cells (Fig 1F). To further measure whether inflammasome activation was increased by BHRF1 expression during the EBV lytic lifecycle, BHRF1 was overexpressed or depleted in the EBV-positive lymphoma cell line P3HR1 (Fig 1G-1H). During the latent stage, neither BHRF1 overexpression nor BHRF1 depletion affected inflammasome activation. However, during lytic replication induced by TPA plus NaB, the cleavage of Caspase-1, IL-1β and IL-18 was increased, and BHRF1 overexpression greatly augmented this increase (Fig 1G). Moreover, their cleavage was dramatically decreased by BHRF1 depletion under lytic induction (Fig 1H). Similarly, BHRF1 overexpression increased inflammasome activation, whereas BHRF1 depletion reduced inflammasome activation in EBV-positive Akata+ cells under IgG induction (S1F-S1G Fig). These results suggest that BHRF1 promotes inflammasome activation during EBV lytic replication.

### BHRF1 induces AIM2 inflammasome activation

**Fig 2:**
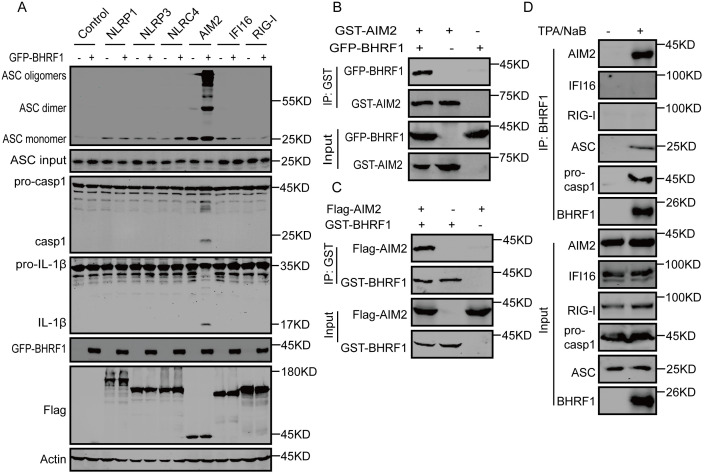
BHRF1 interacts with AIM2 and induces AIM2 inflammasome activation. A. GFP-BHRF1 plasmid or empty vector was co-transfected into HEK293T-CIA cells with empty vector, or Flag-tagged NLRP1, NLRP3, NLRC4, AIM2, IFI16 or RIG-I plasmids for 36 h. The cell extracts were cross-linked with DSS and subjected to analysis of ASC oligomerization by immunoblotting analysis with an anti-ASC antibody as indicated. B. Empty vector or the GST-AIM2 expression plasmid was transfected into HEK293T cells with empty vector or GFP-BHRF1 for 36 h. Cell lysates were immunoprecipitated with GST-affinity beads and then analyzed by western blotting as indicated. C. GST-tagged BHRF1 or empty vector was transfected into HEK293T cells with the empty vector or Flag-AIM2-expressing plasmid for 36 h. The cells were collected, the cell lysates were subjected to immunoprecipitation with GST affinity beads, and the whole-cell lysates and immunoprecipitated complexes were analyzed as indicated. D. P3HR1 cells were left untreated or induced with TPA plus NaB for 48 h, after which the cell lysates were immunoprecipitated with an anti-BHRF1 antibody, and the samples were analyzed by western blotting analysis as indicated.

To further investigate which inflammasomes and signaling pathways are activated by BHRF1 overexpression, 20 inflammasome sensors were individually depleted by shRNA in A549 cells, and inflammasome activation in response to BHRF1 overexpression was measured using pro-IL-1β-DN-Gluc, a Gaussia luciferase-fused and 31aa N-terminal truncated pro-IL-1β protein substrate of Caspase-1. Under HSV-1 infection, AIM2 depletion completely abolished the increase in inflammasome activation induced by BHRF1 overexpression, whereas the depletion of other proteins hardly affected or only slightly affected inflammasome activation (S2A Fig), indicating that BHRF1 promoted AIM2 inflammasome activation. When the six sensors NLRP1, NLRP3, NLRC4, AIM2, IFI16 and RIG-I were separately overexpressed along with ASC, pro-caspase-1 and pro-IL-1β overexpression in HEK293T-CIA cells (HEK293T cell lines with stable pro-caspase-1, pro-IL-1β and ASC overexpression), only AIM2-mediated cleavage of Caspase-1 and IL-1β and ASC oligomerization were induced by BHRF1 overexpression, and other sensor-mediated inflammasome activation was hardly enhanced (Fig 2A), indicating that BHRF1 specifically induced AIM2 inflammasome activation. To further confirm that BHRF1 induces AIM2 inflammasome activation, AIM2 expression was depleted in THP-1 cells. BHRF1 enhanced inflammasome activation in control cells infected with HSV-1, while inflammasome activation was strongly suppressed in AIM2- depleted cells during HSV-1 infection, and BHRF1 overexpression no longer enhanced inflammasome activation (S2B Fig). These results suggest that BHRF1 augmented HSV-1 induced AIM2 inflammasome activation. Similarly, when AIM2 expression was depleted in EBV-positive P3HR1 cells during lytic replication, inflammasome activation was greatly reduced, and BHRF1 did not promote inflammasome activation (S2C Fig). These results suggest that BHRF1 plays the main function in the AIM2 inflammasome activation during the EBV lytic lifecycle.

### BHRF1 recruits the AIM2 inflammasome to mitochondria

**Fig 3:**
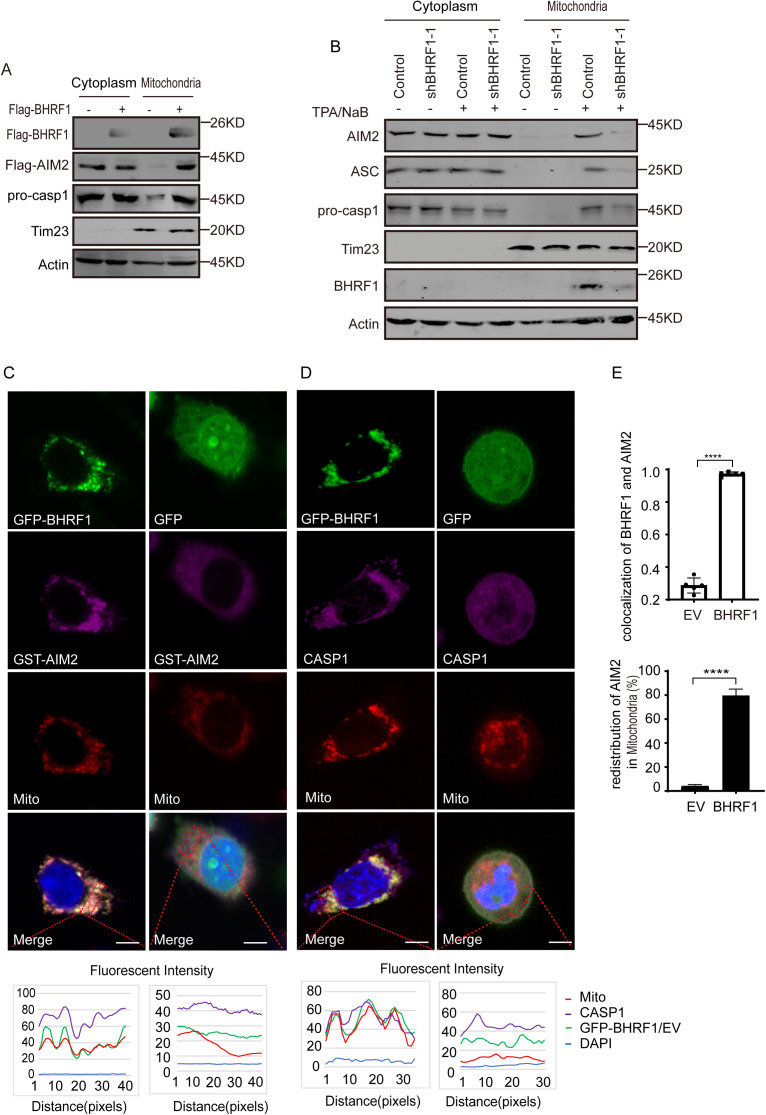
BHRF1 recruits the AIM2 inflammasome to the mitochondrial compartment. A. Empty vector or Flag-BHRF1 plasmid was transfected into HEK293T cells with Flag-AIM2, pro-caspase-1, pro-IL-1β and ASC expression plasmids for 48 h, after which the mitochondrial and cytoplasmic fractions were isolated and analyzed by western blotting analysis as indicated. B. P3HR1 cells were infected with empty or shBHRF1-expressing lentiviruses for 24 h and then left untreated or treated with TPA plus NaB for 48 h. The cytoplasmic and mitochondrial fractions were isolated and analyzed by western blotting. C-D. A549 cells were co-transfected with the GFP-tagged empty vector or the GFP-BHRF1 plasmid and GST-AIM2 (C), or the GFP-tagged empty vector or the GFP-BHRF1 plasmid alone (D) for 36 h. The cells were subsequently stained with MitoTracker Red dye for 30 min and then fixed and stained with an anti-GST antibody (C), an anti-Caspase-1 antibody (D) and a species-matched AlexaFluor 647 secondary antibody. Representative images were visualized by confocal microscopy and are shown. The coefficient of colocalization was determined by qualitative analysis of the fluorescence intensity of the selected areas. Scale bar: 10 μm. E. The quantification of AIM2-BHRF1 colocalization and AIM2 redistribution in mitochondrial compartments were analyzed in five independent images and the percentages of AIM2 protein were calculated and shown. ****, p < 0.0001 by Unpaired t test.

Since BHRF1 is a mitochondrion-localized viral Bcl2 homolog, we wondered whether BHRF1 regulates the mitochondrial distribution of AIM2 and the components of the inflammasome. The mitochondrial and cytoplasmic fractions isolated from control or BHRF1-overexpressing cells were analyzed. Few AIM2 or ASC proteins were distributed in the mitochondrial fraction of control cells, while they were obviously distributed in this fraction in the presence of BHRF1 overexpression (Fig 3A). Similarly, Caspase-1 and IL-1β were strongly enriched in the mitochondrial fraction following BHRF1 overexpression compared with those in control cells (Fig 3A). The distribution of these proteins in the cytoplasmic fraction was not affected by BHRF1 overexpression. Similarly, when the mitochondrial fractions were isolated from EBV-positive cells, the components of the AIM2 inflammasome were enriched in the mitochondrial fraction during EBV lytic replication but not during the latent stage, and depletion of BHRF1 greatly reduced their enrichment in the mitochondrial fraction during the lytic stage (Fig 3B). The distribution of AIM2 and Caspase-1 in the cytoplasm was not affected by BHRF1 overexpression or lytic replication (Fig 3A-3B), suggesting that the majority of AIM2 and Caspase-1 proteins are present in the cytoplasm and that AIM2 maintains its ability to sense cytosolic dsDNA and induce inflammasome activation. Furthermore, the subcellular localization of AIM2 and Caspase-1 in the presence or absence of BHRF1 was analyzed by immunofluorescence. As expected, BHRF1 was localized in the mitochondrial compartment, and both AIM2 and Caspase-1 were enriched and colocalized with BHRF1 in the mitochondrial compartment in BHRF1-expressing A549 cells, whereas they were not distributed in this compartment in control cells (Fig 3C-3D). Similarly, endogenous BHRF1 also colocalized with endogenous AIM2 in P3HR1 cells undergoing EBV lytic replication, whereas BHRF1 cannot be detected in cells under latency (S3A Fig). A quantification of colocalized proteins revealed that a high percentage of AIM2 protein was colocalized with BHRF1 and recruited to the mitochondrial compartment in the context of BHRF1 overexpression, whereas a very low percentage of AIM2 protein was colocalized and redistributed in control cells (Fig 3E). Furthermore, a number of ASC specks were observed in cells undergoing EBV lytic replication compared with latent cells, and BHRF1 also colocalized with these ASC specks (S3C Fig). These results suggest that BHRF1 recruits AIM2 and components of AIM2 inflammasome to the mitochondrial compartment.

### The BHRF1 C-terminal region interacts with the AIM2 HIN domain

To investigate the mechanism of BHRF1-induced AIM2 inflammasome activation, the association between BHRF1 and components of AIM2 inflammasome were evaluated. When BHRF1 and AIM2 were overexpressed in HEK293T cells, immunoprecipitation assays revealed that BHRF1 interacted with AIM2 (Fig 2B-2C). During the EBV lytic lifecycle, endogenous interactions between BHRF1 and AIM2, ASC or Caspase-1 were observed, whereas no interaction with RIG-I or IFI16 was observed (Fig 2D), indicating that BHRF1 binds to the AIM2 inflammasome complex during the EBV lytic lifecycle. To map the binding region, a series of truncations or deletions of BHRF1 and AIM2 were constructed. The AIM2 PYD domain did not bind to BHRF1, and deletion of this domain did not affect the BHRF1-AIM2 interaction; however, deletion of the HIN domain completely abolished this interaction, and AIM2 HIN domain alone was sufficient for this interaction (S4A-S4B Fig), indicating that BHRF1 binds to the AIM2 HIN domain. Alternatively, the deletion of the N-terminal domain, BH3, BH1- or BH2-containing domain in BHRF1 did not abolish the interaction, whereas the truncation of the C-terminal domain Δ157–191 completely abolished it (S4C-S4D Fig). As expected, the truncated construct of the transmembrane (TM) domain in the BHRF1 C-terminus Δ157–191 could not activate the AIM2 inflammasome and the deletion of the aa109–157 region containing BH2 also abolished inflammasome activation (S4E Fig). Moreover, similar to full-length BHRF1, the BHRF1 C-terminal fragments aa157–191 and aa109–191 were able to interact with AIM2 (S4F Fig). However, the BHRF1 C-terminal aa157–191 fragment alone was not able to activate the inflammasome, whereas the aa109–191 fragment containing both the BH2 and TM domains could (S4G Fig). These results suggest that the C-terminal region of BHRF1 is responsible for the interaction of AIM2, but AIM2 inflammasome activation requires an additional region adjacent to this hydrophobic domain.

**Fig 4:**
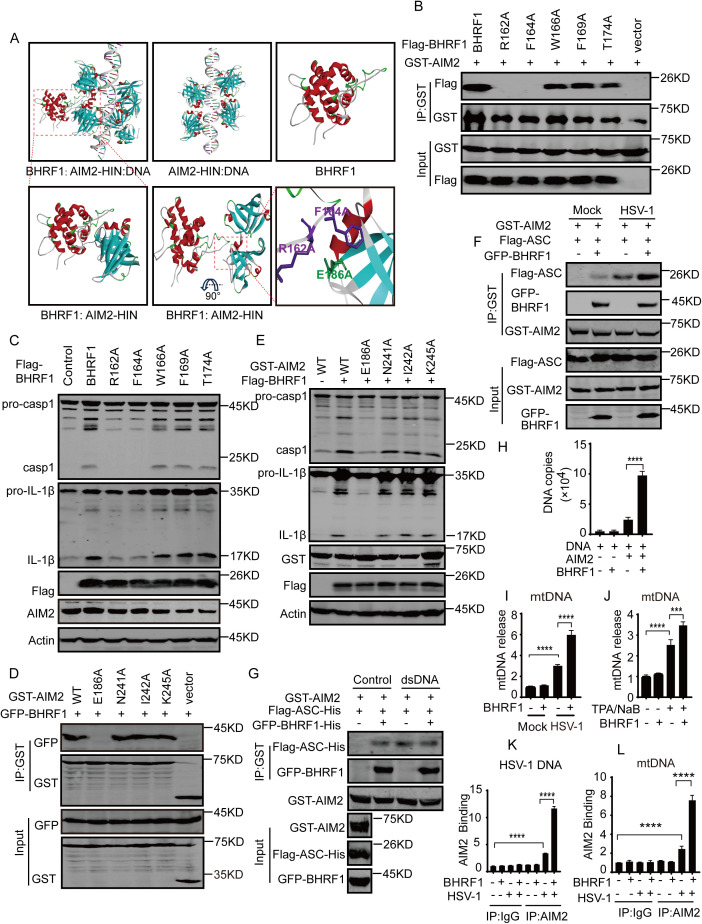
BHRF1 directly binds to AIM2 and promotes the formation of the AIM2 inflammasome. A. The structure of BHRF1 was generated from I-TASSER, and the structure of the AIM2 HIN domain was downloaded from the PDB. The structure files of AIM2-HIN.pdb and BHRF1.pdb were input into the ZDOCK-server as a receptor and a ligand for docking computation, respectively, to generate the ligand–receptor interaction model, and then the BHRF1: AIM2-HIN structure and key residues were analyzed and are shown. B-C. The GST-tagged or Flag- AIM2 plasmid was co-transfected into HEK293T cells (B) or HEK293T-CIA cells (C) with empty vector, Flag-tagged BHRF1 WT or single-point mutated constructs for 36 h. The whole-cell extracts were subjected to immunoprecipitation with GST-affinity beads and western blotting analysis to determine their interaction (B), or to western blotting analysis to determine AIM2 inflammasome activation (C). D-E. The GFP-BHRF1 or Flag-BHRF1 expressing plasmid was transfected into HEK293T cells (D) or HEK293T-CIA cells (E), respectively, with empty vector, GST-AIM2 WT or single-point mutated constructs for 36 h. The cells were collected and lysed, and the cell lysates were subjected to immunoprecipitation with GST-affinity beads followed by western blotting analysis of the BHRF1-AIM2 interaction (D) or western blotting analysis of AIM2-mediated inflammasome activation (E). F. The empty vector or GFP-BHRF1 plasmid was co-transfected into HEK293T cells with GST-AIM2 and Flag-ASC expression plasmids for 24 h, after which the cells were left uninfected or infected with HSV-1 for 12 h. Cell lysates were immunoprecipitated with GST-affinity beads, and the samples were subsequently analyzed by western blotting analysis to determine AIM2-ASC recruitment. G. Purified GST-AIM2, Flag-ASC-His and GFP-BHRF1-His proteins were mixed and incubated in the absence or presence of purified HSV-1 dsDNA overnight at 4 °C. The mixtures were pulled down with GST-affinity beads and subsequently analyzed by western blotting analysis to determine in vitro AIM2-ASC interactions. H. Purified GST-AIM2 and GFP-BHRF1-His proteins alone or together were mixed and incubated with dsDNA overnight at 4 °C. The mixtures were pulled down with GST-affinity beads and AIM2-binding DNA was extracted and analyzed by real-time PCR as indicated. Tukey’s multiple comparisons test. ****, p < 0.0001. I-J. HEK293T-CIA cells were transfected with empty or Flag-BHRF1 expressing plasmids in the presence of AIM2-expressing plasmids for 36 h, and then left uninfected or infected with HSV-1 (MOI = 1) for 12 h (I), or P3HR1 cells were infected with empty or Flag-BHRF1 expressing lentiviruses for 12 h, and then left untreated or induced with TPA plus NaB for 48 h (J), after which the cells were collected and the cytoplasmic fraction was prepared. The mtDNA in cytoplasmic fractions was extracted and analyzed by real-time PCR, and the relative mtDNA release was detected in three independent experiments and are shown. Tukey’s multiple comparisons test. ***, p < 0.0005; ****, p < 0.0001. K-L. AIM2-binding dsDNA was affinity purified from the cytoplasmic fraction as described above (I) with an anti-AIM2 antibody or IgG, extracted and detected by real-time PCR. The relative levels of mtDNA (K) and HSV-1 viral DNA (L) in the precipitated AIM2 complexes were detected in three independent experiments and are shown. Tukey’s multiple comparison test. ****, p < 0.0001.

Next, the AIM2 HIN tertiary structure was downloaded from PDB [8], and the tertiary structure of full-length BHRF1 protein was unavailable, the full-length BHRF1 tertiary structure was predicted and input into the ZDOCK server for computer-based simulation and docking. The structures matching the AIM2 HIN domain and BHRF1 tertiary structure were docked, a loop of the BHRF1 C-terminal fragment was inserted into the pocket between the AIM2 HIN OB1 and OB2 subdomains but not the interface of AIM2 HIN binding to dsDNA or the gap between two AIM2 HIN domains, and several residues in the BHRF1 C-terminal loop and AIM2 HIN interfaces were anchored to each other, as revealed by the Discovery Studio tool (Figs 4A and S5A). To further understand the BHRF1-AIM2 structure and mechanism of BHRF1-mediated AIM2 activation, the key residues in the BHRF1 or AIM2 binding interfaces were separately mutated. Two single-point R162A and F164A mutations in BHRF1 abolished the interaction with AIM2 (Fig 4B) and dramatically reduced the ability to activate the AIM2 inflammasome (Fig 4C). Moreover, a single-point AIM2 E186A mutant did not bind to BHRF1 (Fig 4D) and could not be activated by BHRF1 overexpression (Fig 4E), whereas other single-point mutations in the BHRF1 or AIM2 constructs did not affect binding or activity in BHRF1-mediated AIM2 inflammasome activation. However, the AIM2 E186A mutation minimally affected the ability of AIM2-AIM2 self-interaction and inflammasome activation during HSV-1 infection (S5B-S5C Fig), suggesting that BHRF1 induced AIM2 inflammasome activation through a new mechanism.

In addition, we determined whether inflammasome activation affects BHRF1 function in preventing apoptosis and autophagy. The wild-type BHRF1, R162A or F164A construct was overexpressed in BHRF1 KO HNE-1–2089 cells that harbor BHRF1-null EBV Bacmid p2089 genome and lack BHRF1 expression, followed by the induction of apoptosis or autophagy. Both wild-type and mutated BHRF1 overexpression suppressed the caspase-3 and caspase-8 cleavage induced by cisplatin treatment (S5D Fig), suggesting that BHRF1 R162A or F164A mutation did not affect its antiapoptotic function. They similarly induced an LC3-II/LC3-I shift and decreased p62 levels (S5E Fig), indicating that the BHRF1 R162A or F164A mutation did not alter its autophagy-inducing function. Alternatively, the BHRF1 G99A or R100D mutants, which significantly reduced the inhibition of cell death, still induced inflammasome activation (S5F Fig). These results suggest that the function of BHRF1 in inflammasome activation is independent of its antiapoptotic and autophagic effects.

### BHRF1 directly enhances AIM2-ASC recruitment and AIM2-dsDNA recognition

To further understand the mechanism of BHRF1-mediated AIM2 inflammasome activation, the polymerization of AIM2 and AIM2-ASC recruitment were investigated in the absence or presence of BHRF1. The recruitment of ASC by AIM2 was increased in the presence of BHRF1 overexpression in untreated cells and cells during HSV-1 infection or under poly(dA:dT) stimulation (Figs 4F and S6A). These results suggest that BHRF1 induces the assembly of AIM2 inflammasome complexes in cells. To further determine whether AIM2-AIM2 polymerization or the AIM2-ASC interaction was directly induced by BHRF1 in vitro, purified AIM2, ASC and BHRF1 proteins were prepared. When GST-tagged AIM2 and Flag-tagged AIM2 proteins were mixed together in vitro in the absence or presence of purified dsDNA, the BHRF1 protein alone did not induce the AIM2-AIM2 interaction, whereas the purified dsDNA did, indicating that BHRF1 did not trigger AIM2-AIM2 polymerization in the same way as dsDNA did (S6B Fig). However, when GST-tagged AIM2 was mixed with Flag-tagged ASC protein under the same conditions, the pull-down assays clearly revealed that BHRF1 alone promoted the AIM2-ASC interaction in the absence of dsDNA, and the interaction was comparable to that in the presence of dsDNA (Fig 4G). Furthermore, when the AIM2 protein was mixed with dsDNA in vitro in the absence or presence of the BHRF1 protein, BHRF1 itself did not bind to dsDNA, whereas the dsDNA binding of AIM2 protein was increased in the presence of the BHRF1 protein (Fig 4H). To investigate AIM2-dsDNA binding in the absence or presence of BHRF1 expression, mitochondrial DNA (mtDNA) in the cytoplasm was extracted and analyzed, and the results revealed that HSV-1 infection and EBV lytic reactivation induced mtDNA release and that BHRF1 overexpression alone did not induce mtDNA release in control cells but greatly increased mtDNA release in HSV-1-infected cells or EBV lytic cells (Fig 4I-4J). Consequently, AIM2 binding to mtDNA and viral DNA was increased by BHRF1 overexpression in HSV-1 infected cells but not in uninfected cells (Fig 4K-4L). These results suggest that BHRF1 binds to AIM2 and promotes AIM2 binding to dsDNA and the ASC protein, likely by disrupting the intramolecular autoinhibition of AIM2 PYD-HIN.

### BHRF1 promotes EBV lytic replication through the AIM2-Caspase-1 axis

**Fig 5:**
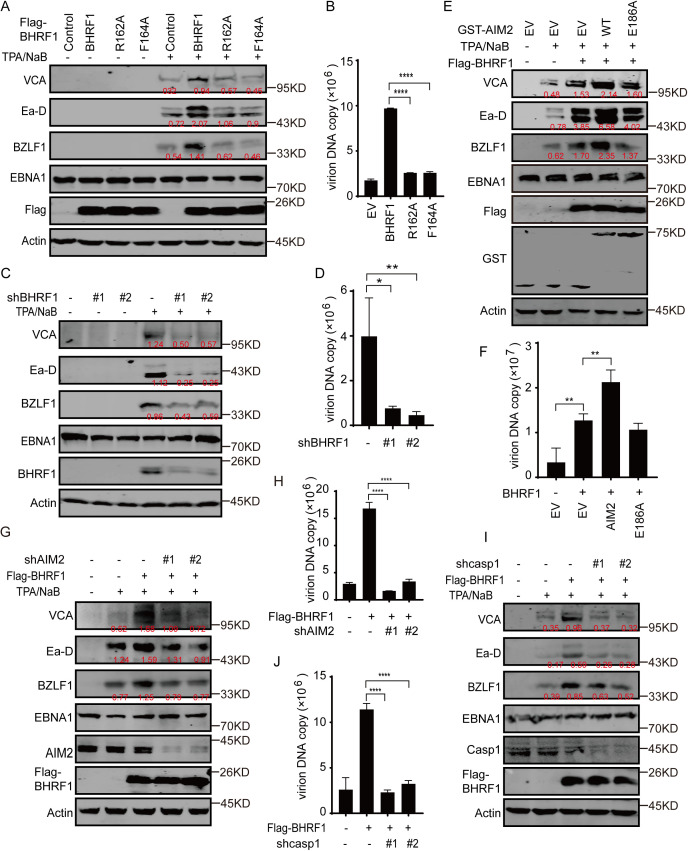
AIM2 and Caspase-1 activation are essential for BHRF1-promoted EBV lytic replication. A-D. P3HR1 cells were infected with empty, BHRF1 wild-type, R162A- or F164A-expressing lentiviruses (A-B), or scramble shRNA or shBHRF1-expressing lentiviruses (C-D) for 24 h and then left untreated or treated with TPA plus NaB for lytic induction. Forty-eight hours later, the cell lysates were analyzed by western blotting analysis of viral gene expression (A, C). Ninety-six hours later, the supernatants were collected, and the extracellular virion DNA was extracted and analyzed by real-time PCR (B, D). The data are shown as the mean ± SD (n = 3), Tukey’s multiple comparison test. *, p < 0.05; **, p < 0.01; ****, p < 0.0001. E-F. P3HR1 cells were transiently infected with empty, GST-AIM2 wild-type or E186A-expressing lentiviruses. The cells were then left untreated or treated with TPA plus NaB. After 72 h, the cells were collected and the cell extracts were analyzed by western blotting (E). The supernatants were collected after 96 h and extracellular virion DNA was extracted and analyzed by real-time PCR (F). Data are shown as the mean ± SD (n = 3), Tukey’s multiple comparison test. **, p < 0.01. G-J. P3HR1 cells were cotransfected with empty or BHRF1-expressing lentiviruses and empty or shAIM2-expressing lentiviruses (G-H), or empty or shCaspase-1-expressing lentiviruses (I-J) for 24 h, after which the cells were left untreated or treated with TPA plus NaB. After 72 h, the cells were collected and the cell extracts were analyzed by western blotting for viral gene expression (G, I). After 96 h, the extracellular virions were collected and the virion DNA was extracted and analyzed for virion production (H, J). Data are shown as the mean ± SD (n = 3), Tukey’s multiple comparison test. ****, p < 0.0001.

Because BHRF1 is a lytic protein in the EBV lifecycle, the function of BHRF1-inflammasome activation during EBV lytic replication was further investigated. When the BHRF1 WT, R162A or F164A construct was overexpressed in P3HR1 cells, chemical-induced expression of both the early gene BZLF1 and the late lytic genes VCA and Ea-D was strongly enhanced by BHRF1 WT overexpression, but not by BHRF1 R162A or F164A overexpression (Figs 5A and S7A). Consequently, virion production was increased by BHRF1 WT overexpression but not by BHRF1 R162A or F164A overexpression (Fig 5B). However, the BHRF1 G99A or R100D mutation increased lytic gene expression and virion production to a level equal to that of the BHRF1 wild type (S7B-S7C Fig), indicating that the antiapoptotic function of BHRF1 is not involved in the process of EBV lytic replication. In contrast, when BHRF1 expression was depleted in P3HR1 cells, lytic gene expression dramatically decreased (Fig 5C). As a result, virion production was reduced by BHRF1 depletion (Fig 5D). Latent gene expression of EBNA1 was not affected by either BHRF1 overexpression or depletion. Similarly, BHRF1 WT overexpression increased while BHRF1 R162A or F164A overexpression did not affect the levels of lytic gene expression or virion production, and BHRF1 depletion reduced their levels in Akata+ cells under IgG induction (S7D-S7G Fig). The same effects were also observed in P3HR1 cells under lytic induction with ectopic BZLF1 overexpression (S7H-S7I Fig). These results suggest that BHRF1-mediated inflammasome activation is required for EBV lytic replication.

Furthermore, the functions of wild-type or mutant AIM2 in EBV lytic replication were measured. Wild type AIM2 overexpression increased viral lytic gene expression and AIM2 depletion decreased it, whereas mutant E186A promoted neither latent nor lytic gene expression in EBV life cycle (Figs 5E and S8A-S8C); subsequently wild type AIM2 increased EBV virion production, and the AIM2 E186A mutant lost the majority of its activity in virion production (Fig 5F). The results show that a moderate level of AIM2 expression strongly increased the replication of the viral DNA genome and virion production, whereas a high level of AIM2 expression weakly increased viral DNA replication and virion production (S8D-S8E Fig), indicating that endogenous and low AIM2 expression results in a pro-viral function during EBV lytic replication, whereas high AIM2 overexpression triggers an additional antiviral effect to counteract this function.

To further determine the role of BHRF1-mediated AIM2 inflammasome activation in lytic replication, AIM2 expression was depleted in control or BHRF1-overexpressing cells. In control cells, AIM2 depletion reduced EBV lytic gene expression and virion production but did not affect latent gene expression (S8F-S8G Fig). In cells overexpressing BHRF1, the BHRF1-mediated increase in lytic gene expression was reduced by AIM2 depletion (Fig 5G). Consequently, AIM2 depletion completely abolished this increase in virion production in the presence of BHRF1 overexpression (Fig 5H). Similarly, Caspase-1 depletion did not affect latent gene expression but decreased late lytic gene expression and strongly decreased BHRF1-induced virion production (Fig 5I-5J). These results suggest that BHRF1 promotes EBV lytic replication through AIM2 inflammasome activation.

### BHRF1-AIM2 activation promotes lytic replication without causing mitochondrial dysfunction

**Fig 6:**
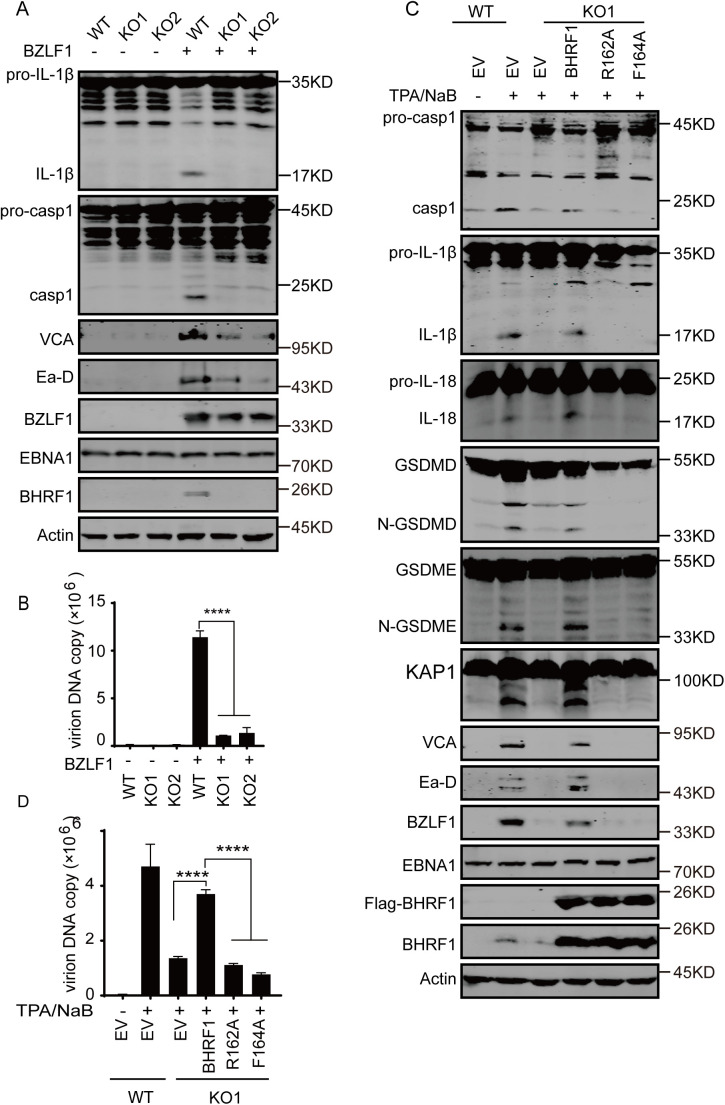
Reintroduction of BHRF1 and vBcl2 expression restores AIM2 inflammasome activation and EBV lytic replication in BHRF1-null EBV infected cells. A-B. Wild-type or BHRF1-null HNE-1-2089 cells were transiently transfected with BZLF1 expression plasmid to induce lytic replication. After 48 h, the cells were collected and the cell lysates were analyzed by western blotting analysis as indicated (A). Extracellular virion DNA was analyzed by real-time PCR after 96 h (B). Data are shown as the mean ± SD (n = 3), Sidak’s multiple comparisons test. ****, p < 0.0001. C-D. BHRF1-null HNE-1-2089 cells were infected with Flag-tagged control, BHRF1 wild-type, R162A- or F164A- expressing plasmids for 24 h and then left untreated or treated with TPA plus NaB. After 48 h of induction, the cell lysates were analyzed by western blotting analysis as indicated (C). Ninety-six hours later, the extracellular virion DNA was analyzed by real-time PCR (D). Data are shown as the mean ± SD (n = 3), Sidak’s multiple comparisons test. ****, p < 0.0001.

To further confirm that BHRF1 promotes lytic replication through AIM2 inflammasome activation, BHRF1-null cells and viruses were generated through CRISPR/Cas9-mediated mutagenesis in HNE1 cells harboring the EBV Bac-p2089 genome. When BHRF1 was knocked out, the cleavage of Caspase-1 and IL-1β was abolished under lytic induction of BZLF1 overexpression, indicating that inflammasome activation was dramatically suppressed by BHRF1 deficiency during lytic replication (Fig 6A). Consequently, lytic gene expression and virion production were similarly suppressed in BHRF1-null cells compared with control cells during BZLF1-induced lytic replication (Fig 6A-6B). When BHRF1 WT, R162A or F164A was reintroduced into BHRF1-null cells, BHRF1 WT restored the cleavage of Caspase-1, IL-1β and IL-18 during lytic replication, whereas neither R162A nor F164A expression restored this cleavage (Fig 6C). Similarly, BHRF1 WT overexpression restored lytic gene expression and virion production, whereas neither BHRF1 R162A nor F164A overexpression did (Fig 6C-6D). Interestingly, KAP1 cleavage was observed during EBV lytic replication in control cells, but was undetectable in BHRF1-null cells under lytic induction, and BHRF1 WT overexpression restored KAP1 cleavage, whereas neither BHRF1 R162A nor F164A overexpression did (Fig 6C), indicating that BHRF1-mediated inflammasome activation removes KAP1 repression to facilitate viral transcription and replication. These results confirmed that BHRF1-mediated inflammasome activation is essential for EBV lytic gene expression and replication.

Since AIM2 activation can trigger PANoptosis during dsDNA viral and bacterial infection [10], we further determined whether BHRF1-mediated AIM2 inflammasome activation affects cell death during EBV lytic replication. In BHRF1-null EBV Bac-p2089-harboring HNE1 cells, reintroduction of BHRF1 WT but not BHRF1 R162A or F164A overexpression induced GSDMD and GSDME cleavage following inflammasome activation (Fig 6C). Consequently, a low level of PI-positive cells was induced by BHRF1 WT but not by BHRF1 R162A or F164A expression (S9A-S9B Fig), indicating that a low basal level of GSDMD pore and pyroptosis were induced by BHRF1 expression during the lytic lifecycle. Similarly, mtDNA release was increased in cells expressing BHRF1 WT but not BHRF1 R162A or F164A under lytic induction (S9C Fig), suggesting that the GSDME pore in the mitochondrial membrane and mtDNA release were increased by BHRF1 expression during EBV lytic stage. The intensity of mitotracker staining was decreased in WT cells but not in BHRF1 KO cells during EBV lytic replication, and overexpression of BHRF1 WT, R162A or F164A decreased it in KO cells under lytic induction (S9D Fig), suggesting that these BHRF1 proteins might stimulate the mitophagy as previously described [30]. However, the number and membrane potential of mitochondria were barely affected by the presence of either WT or mutated BHRF1 during the lytic lifecycle (S9E Fig). Importantly, no cleavage of caspase-3 was observed in presence of any overexpression during lytic replication (S9F Fig). Additionally, neither BHRF1 WT nor BHRF1 R162A or F164A overexpression induced the phosphorylation of RIPK3, the initiating kinase of necroptosis (S9G Fig). Importantly, BHRF1 WT, R162A and F164A did not increase LDH release from cells during EBV lytic cycle while decreased LDH release under cisplatin treatment, these results suggest that BHRF1-induced AIM2 inflammasome activation hardly induced cell death (S9H Fig). These results suggest that BHRF1-induced AIM2 inflammasome activation does not cause obvious mitochondrial dysfunction, apoptosis or necroptosis during lytic replication.

**Fig 7:**
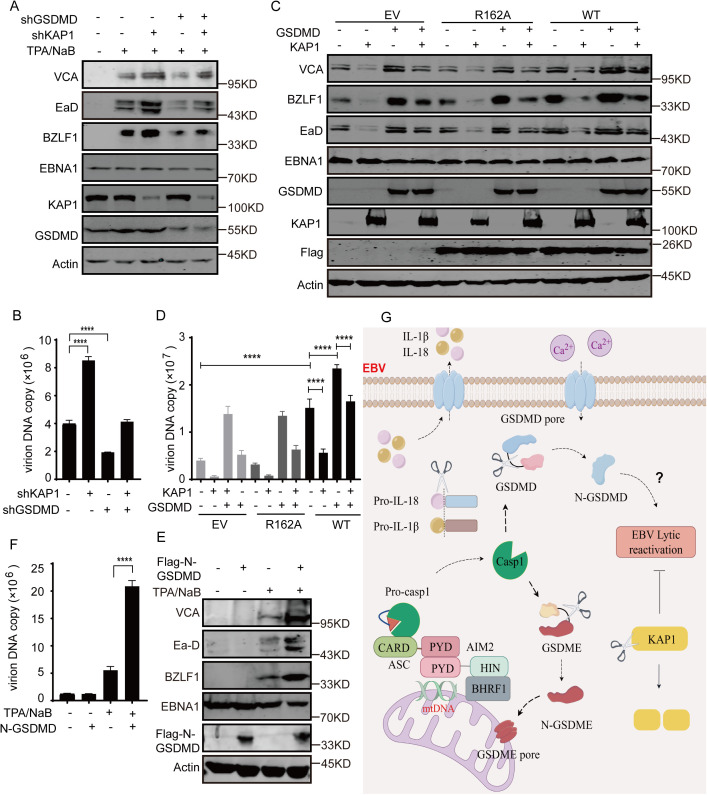
KAP1 and GSDMD play essential roles in EBV lytic replication. A-B. P3HR1 cells were infected with scramble, shKAP1 or shGSDMD-expressing lentiviruses alone or together for 24 h and then left untreated or treated with TPA plus NaB. The cell extracts were analyzed by western blotting after 48 h of induction (A). The supernatants were collected after 96 h of induction, and extracellular virion DNA was extracted and analyzed by real-time PCR (B). Data are shown as the mean ± SD (n = 3). Tukey’s multiple comparison test. ****, p < 0.0001. C-D. P3HR1 cells were infected with empty, BHRF1 WT or R162A-expressing lentiviruses with empty, KAP1 or GSDMD-expressing lentiviruses alone or in combination for 24 h and then left untreated or treated with TPA plus NaB. Then viral gene expression (C) and virion production (D) were analyzed as described above. The data are shown as the mean ± SD (n = 3). Tukey’s multiple comparison test. ****, p < 0.0001. E-F. P3HR1 cells were infected with empty, N-GSDMD expressing lentiviruses for 24 h and then left untreated or treated with TPA plus NaB for lytic induction. Forty-eight hours later, the cell lysates were analyzed by western blotting analysis of viral gene expression (E). Ninety-six hours later, the supernatants were collected, and the extracellular virion DNA was extracted and analyzed by real-time PCR (F). The data are shown as the mean ± SD (n = 3). Tukey’s multiple comparison test. *, P < 0.05; ****, p < 0.0001. G. Diagram and pathways of AIM2 inflammasome activation during EBV lytic cycle. During EBV lytic replication, BHRF1 interacts with AIM2, recruits the AIM2 inflammasome to the mitochondrial compartment, and subsequently activates the AIM2 inflammasome in cooperation with viral dsDNA or mtDNA. As a result, active caspase-1 cleaves the substrates pro-IL-1β, pro-IL-18, GSDMD, GSDME and KAP1 to release mature IL-1β and IL-18 and promote EBV lytic reactivation.

Finally, the roles of KAP1 and GSDMD cleavage during EBV lytic replication were investigated. Depletion of GSDMD expression alone significantly reduced EBV lytic gene expression and virion production (Figs 7A-7B and S10A-S10B), while depletion of KAP1 expression alone greatly increased the expression of these genes (Figs 7A-7B and S10C-S10D); consequently, double GSDMD and KAP1 depletion together resulted in a compromised phenotype of viral gene expression and virion production (Fig 7A-7B), probably because the level of cleaved N-GSDMD fragments decreased following GSDMD depletion and KAP1 depletion attenuated epigenetic suppression similar to KAP1 cleavage. Similarly, EBV lytic gene expression and virion production were increased by GSDMD overexpression alone but greatly decreased by KAP1 overexpression alone (Fig 7C-7D), and the levels were also compromised under combined overexpression together (Fig 7C-7D). Interestingly, these effects were augmented by BHRF1 wild type overexpression but were minimally affected by BHRF1 R162A overexpression (Fig 7C-7D). To confirm the function of GSDMD cleavage in EBV lytic replication, the cleaved N-terminal GSDMD fragment was overexpressed, and increased EBV lytic gene expression and virion production were observed in these cells compared with the control cells under lytic induction (Fig 7E-7F), confirming that the N-terminal GSDMD fragment has a prolytic effect after Caspase-1-mediated GSDMD cleavage in cells undergoing lytic replication. These findings suggest that both KAP1 cleavage and GSDMD cleavage are important for effective EBV lytic replication.

Taken together, our findings reveal that BHRF1 interacts with AIM2, recruits AIM2 inflammasome complexes to the mitochondrial compartment and then induces AIM2 inflammasome activation to promote EBV lytic replication through KAP1 and GSDMD cleavage (Fig 7G), which represents an unrecognized subversion of AIM2 inflammasome activation during EBV lytic replication.

## Discussion

Herpesvirus infection activates the AIM2 inflammasome, which triggers cell pyroptosis and PANoptosis [10], and several viral proteins suppress AIM2 inflammasome activation to promote immune evasion and lytic replication [22,23]. As AIM2 is a predominantly cytosolic dsDNA sensor, how the AIM2 inflammasome is finely tuned by viral products during the lytic replication of oncogenic herpesviruses and whether viral proteins can trigger AIM2 inflammasome activation largely remain unknown. In the present study, we revealed that EBV BHRF1 bound to AIM2 and promoted the AIM2 inflammasome activation during lytic replication. BHRF1 deficiency strongly decreased inflammasome activation during lytic replication, and wild-type BHRF1 overexpression restored inflammasome activation, whereas neither BHRF1 R162A nor F164A overexpression interacted with nor induced the AIM2 inflammasome activation. These results suggest that EBV BHRF1 plays an essential function in the AIM2 inflammasome activation during lytic replication.

We investigated BHRF1 expression and inflammasome activity during the entire EBV lifecycle in P3HR1 cells and found that BHRF1 was expressed at a low level during EBV latency without obvious inflammasome activation and that BHRF1 expression and inflammasome activity were greatly increased at the late stage of lytic replication (S1A Fig). BHRF1 knockdown or knockout greatly decreased inflammasome activity during late lytic stage (Figs 1F, 6A and S1G). Neither low BHRF1 expression during latency nor BHRF1 overexpression alone is sufficient to induce inflammasome activation, while high BHRF1 expression cooperates with viral dsDNA to enable AIM2 inflammasome activation during the EBV lytic cycle. These results suggest that BHRF1 strongly promotes inflammasome activation during late EBV lytic replication.

AIM2 inflammasome activation requires AIM2 polymerization, ASC recruitment and priming of inflammasome components. The intramolecular PYD-HIN interaction is responsible for AIM2 autoinhibition and inactivation, and dsDNA recognition and binding of the HIN domain trigger a change in AIM2 conformation, which consequently induces AIM2-AIM2 polymerization along the dsDNA chain. Then, ASC is recruited through interaction with the AIM2 PYD domain [8,9], which finally forms the complexes of AIM2 inflammasome with pro-caspase-1 and substrates. Our study revealed that the BHRF1 C-terminal region bound to the AIM2 HIN domain and that BHRF1 directly induced the AIM2-ASC interaction; however, BHRF1 was not able to trigger AIM2-AIM2 polymerization in the absence of dsDNA when the three kinds of purified proteins were mixed together in vitro. These results suggest that BHRF1 binds to the AIM2 HIN domain and subsequently promotes dsDNA binding and ASC recruitment, probably through disrupting the intramolecular PYD-HIN interaction and autoinhibition. However, the interaction of the BHRF1 C-terminal region with AIM2 might not be sufficient to activate the AIM2 inflammasome because deletion of the 109–157 region in BHRF1 did not affect the interaction with AIM2 but abolished AIM2 inflammasome activation (S4D-S4E Fig). Moreover, a short BHRF1 C-terminal fragment did not activate the AIM2 inflammasome even though it binds to AIM2 (S4F-S4G Fig). The additional region or the unique topology of BHRF1 in the outer mitochondrial membrane is likely important for triggering conformational changes in AIM2 and the AIM2-ASC interaction.

BHRF1 is mainly localized in the outer mitochondrial membrane and play antiapoptotic roles during lytic replication. As a result, the AIM2 inflammasome complex is recruited to the mitochondrial compartment through BHRF1 expression during the EBV lytic lifecycle (Fig 3). Why is the AIM2 inflammasome recruited to mitochondria during lytic replication? Our explanation is that EBV primary infection and lytic reactivation can induce mitochondrial dysfunction and oxidative stress [30,32], which cause mitochondrial mtDNA release. The AIM2 inflammasome, which is recruited to the outer surface of mitochondria by BHRF1, is involved in mtDNA release and easily recognizes mtDNA to augment inflammasome activation. Like mitochondria serve as platforms for inflammasome activation, MAVS recruits the NLRP3 inflammasome to the mitochondrial compartment, where ROS are produced [33].

Although AIM2 inflammasome activation can induce PANoptosis through the recruitment of pro-caspase-1, pro-caspase-8 and RIPK3 [10], caspase-8 and caspase-3 cleavage were not induced by BHRF1 expression during EBV lytic replication, indicating that BHRF1-mediated AIM2 inflammasome activation does not induce apoptosis, probably because of the antiapoptotic function of BHRF1. Similarly, even though the AIM2 inflammasome was recruited to the mitochondrial compartment, mitochondrial dysfunction was not induced by BHRF1 wild-type, R162A or F164A BHRF1 overexpression. However, the cleavage of Caspase-1, IL-1β, IL-18, GSDMD and GSDME was observed in BHRF1 wild-type but not R162A or F164A expressing cells during lytic replication. Consequently, a low basal level of pyroptosis and mtDNA release was observed during the lytic lifecycle in the presence of wild-type but not mutated BHRF1 (S9A-S9C Fig). These results suggest that BHRF1-mediated AIM2 inflammasome activation does not cause obvious cell death during lytic replication, probably because the strength of inflammasome activation is finely tuned by viral products below the threshold for pyroptosis or because BHRF1 preferentially controls the selection of substrates for the AIM2 inflammasome to facilitate proinflammatory responses and lytic replication.

Although AIM2 inflammasome activation is induced by viral DNA and mtDNA during herpesviruses infection, it is inhibited by different viral proteins, including HSV-1 VP22 [22], HCMV pUL83 [34], MCMV M84 [35] and KSHV SOX [23], which evade AIM2-related antiviral responses and pyroptosis to ensure effective viral infection and propagation. However, activation of AIM2 inflammasome is induced by EBV BHRF1 during EBV lytic cycle. In fact, inflammasome activation serves as an important activator of EBV lytic replication, and the inhibition of inflammasome activation decreases EBV lytic reactivation from latency [24,36]. Our study revealed that BHRF1-induced AIM2 inflammasome activation does not restrict but rather promotes EBV lytic replication. AIM2 overexpression positively affects lytic gene expression at the late stage, and promotes viral DNA replication and virion production, and the BHRF1 wild-type but not the R162A or F164A construct mediates KAP1 cleavage during lytic replication, similar to the pro-viral function of NLRP3 inflammasome activation during the EBV lytic lifecycle [24]. Similarly, GSDMD cleavage is increased by BHRF1-AIM2 axis and is required for lytic gene expression and virion production, likely leading to the formation of a pore in the plasma and mitochondrial membrane to cause Ca^2+^ leakage and mitochondrial ROS release [37,38], which can trigger EBV lytic reactivation. Consequently, KAP1 and GSDMD cleavage are induced through both AIM2 and the NLRP3 inflammasome to promote transcriptional activation. In addition, Caspase-1 cleaves viral proteins such as BPLF1 to promote viral replication, assembly and release [39]. These results suggest that the activation of AIM2 inflammasome and Caspase-1 is important for facilitating late EBV lytic replication rather than acting as a cellular restriction factor.

Notably, BHRF1 overexpression alone did not trigger inflammasome activation or initiate lytic gene expression (Figs 1E and 5A) but increased the expression of both genes after lytic induction. Similarly, overexpression of the cleaved GSDMD N-terminal fragment alone is insufficient to initiate lytic gene expression but promotes lytic replication under lytic reactivation (Fig 7E-7F), suggesting that inflammasome activation–mediated GSDMD cleavage does not initiate but rather facilitates lytic replication. Furthermore, another substrate, KAP1, is cleaved by Caspase-1 (Fig 6C), and full-length KAP1 has a suppressive effect on lytic gene expression and virion production through epigenetic suppression (Fig 7A-7D). Thus, both KAP1 and GSDMD cleavage can suppress the expression of all IE and early and late genes via a feedback mechanism. These findings indicate that the BHRF1-AIM2-Caspase-1 axis augments lytic replication through multiple mechanisms after the lytic cycle has been initiated by lytic induction.

Studies have revealed that human oncogenic herpesviruses employ multiple strategies to finely tune the type, strength and timing of inflammasome activation. During the KSHV lytic cycle, NLRP1 inflammasome is activated by the viral IE protein ORF45 but suppressed by the late viral protein ORF63 [21,40]. Similarly, NLRP3 and RIG-I inflammasome activation are suppressed by BILF1 and BRLF1 during EBV lytic cycle [19,41], respectively, whereas the AIM2 inflammasome is activated by BHRF1. Our preliminary screening of viral ORF expressing library revealed that EBV encode more inhibitors and activators of inflammasome activation [19,23], which represent the unrecognized subversions of inflammasome activation during lytic replication. It is conceivable that viral products selectively activate inflammasomes and suppress antiviral responses at early stage to facilitate effective lytic replication through KAP1 and GSDMD cleavage, while they cooperatively limit the strength and outcomes of inflammasome activation at later stage to prevent inflammatory damage and pyroptosis. Thus, we conclude that inflammasome activation is finely and abundantly regulated during the EBV lytic cycles, to ensure the effective evasion or hijacking of inflammasome activation for infection and replication.

Furthermore, BHRF1 is also transiently expressed during primary EBV infection, and AIM2 is upregulated after infection [42,43]; a similar BHRF1-AIM2 interaction and AIM2 inflammasome activation might occur during early EBV de novo infection and play important roles in immune and inflammatory responses. Studies have revealed that IL-1β/IL-18 can induce T and NK cell activation and killing [19] and that IL-1β recruits neutrophils to control tumor growth and prolong survival in EBV-induced nasopharyngeal carcinoma model animals [44]. During the prelatent stage of primary EBV infection, EBV transiently expresses BHRF1 to promote AIM2 inflammasome activation in cooperation with viral genomic dsDNA; consequently, increased IL-1β/IL-18 secretion triggers antiviral and antitumor immune and inflammatory responses, serving as host restriction factors for the establishment of latency and persistent infection.

Compared with AIM2 inflammasome activation, BHRF1 expression has a more profound functional effect on EBV lytic replication, and promotes both lytic gene expression and virion production (Fig 5A-5D), indicating the additional function of BHRF1 during EBV lytic replication. In fact, BHRF1 enhances autophagy and suppresses innate immune responses [30], both of which facilitate EBV lytic gene expression and lytic replication. This function may be conserved in human oncogenic herpesviruses, because KSHV vBcl2 is also essential for KSHV lytic gene expression and the assembly of virion particles [28,29]; however, the N-terminal region, but not the C-terminal region, is responsible for this function [45].

In conclusion, our study revealed that BHRF1 plays an important function in AIM2 inflammasome activation during EBV lytic replication. BHRF1 binds to the AIM2 HIN domain and promotes the AIM2 inflammasome activation, likely by disrupting the intramolecular autoinhibition of AIM2 to promote dsDNA binding and ASC recruitment. This leads to the recruitment of AIM2, ASC and pro-caspase-1, which assemble the AIM2 inflammasome complex, in the mitochondrial compartment near the site of mtDNA release, subsequently promoting the cleavage of substrates such as KAP1 and GSDMD to facilitate late EBV lytic replication. Our findings indicate that Epstein–Barr virus employs a novel strategy to hijack AIM2 inflammasome activation and promote lytic replication through Caspase-1-dependent substrates.

## Materials and methods

### Cells, antibodies and chemicals

HEK293T cells were used for detection of protein-protein interaction, THP1 and HEK293T-CIA cells (HEK293T cell line stably expressing pro-Caspase-1, pro-IL-1β and ASC) were used for the assays of inflammasome activation, A549 cells were used for screening of inflammasome activation, EBV-positive P3HR1, Akata+ and HNE-1–2089 (stable HNE-1 cells harboring EBV bacmid p2089) cells were used for detection of EBV gene expression and lytic replication. HEK293T, HEK293T-CIA, HNE-1–2089 and A549 cell lines were maintained in our laboratory and cultured in DMEM supplemented with 10% fetal bovine serum (FBS, Bio-Channel) and 1% antibiotics (penicillin‒streptomycin solution, NCM biotech). The lymphoma cell lines P3HR1, Akata + , BJAB and THP-1 were cultured in RPMI 1640 medium supplemented with 10% FBS and 1% antibiotics. Wild-type HSV-1 KOS strain was maintained in our laboratory and used to induce AIM2 inflammasome activation.

The following antibodies were used in this study: anti-caspase-1 (A0964),anti-caspase-8 (A0215) were purchased from ABclonal; anti-IL-1β (GTX130021), anti-EBV VCA (GTX42976) were purchased from GeneTex; anti-AIM2 (20590–1-AP), anti-GSDMD (20770–1-AP), anti-IL-18 (10663–1-AP) and anti-ASC (10500–1-AP) were purchased from Proteintech; anti-GSDME (AB215191) was purchased from Abcam; anti-Flag (M185-3 L) was purchased from MBL; anti-BHRF1 (5E270), anti-BZLF1 (sc-53904), anti-BMRF1 (sc-58121) and anti-EBNA1 (sc-57719) were purchased from Santa Cruz; anti-KAP1 (RT1210–2), anti-Actin (EM21002), anti-RIPK1 (ER1901) and anti-GST (ET1611–47) were purchased from HuaAn Biotechnology; anti-GFP (RM1008) was purchased from Ray Antibody Biotech; anti-p-RIPK3 (D6W2T), anti-caspase-3 (#9662) and anti-cleaved caspase-3 (#9664) were purchased from Cell Signaling Technology; anti-ICP0 antibody (ab6513) was purchased from abcam. Goat anti-human IgG (H0111-6) was purchased from Zhenglong Biochem; Goat anti-mouse IgG IRDye680RD (C90710-09), Goat anti-mouse IgG IRDye800RD (926–32210), Donkey anti-Rabbit IgG IRDye680LT (926–68023) and goat anti-rabbit IgG IRDye800CW (C80925-05) secondary antibodies were purchased from LI-COR.

Human IL-1β ELISA Kits (88-7261-77) were purchased from Thermo Fisher. Human IL-18 ELISA Kits (Elabscience, E-EL-H0253c) were purchased from YONG JIN Biotechnology. 12-O-Tetradecanoyl-phorbol-13-acetate (TPA) and sodium butyrate (NaB) were purchased from Sigma-Aldrich. Propidium iodide (PI, KGA214) was purchased from KeyGen Biotech. Rhodamine123 (HY-D0816) was purchased from MedChemExpress. Cisplatin (IC0440) was purchased from Solarbio.

### shRNA ang sgRNA

All shRNAs were inserted into the pLKO.1 lentivirus expression vector, and CRISPR-Cas9 sgRNAs were inserted into the lentiCRISPR V2 vector. The following BHRF1 shRNA and sgRNA sequences were used:

shBHRF1–1: 5’-TGGATGGTTGGATTCATCAAC-3’;
shBHRF1–2: 5’-ATGGTTGGATTCATCAACAGG-3’;
sgBHRF1#1: 5’-GGTTGGATTCATCAACAGGG-3’;
sgBHRF1#2: 5’- GATGGTTGGATTCATCAACA-3’.

The shRNA used for inflammasome-forming sensors and proteins were described previously [19], and all the sequences of shRNA were listed in S1 Table.

### Establishment of WT and BHRF1 knockout (KO) cell lines

HNE-1 cells were transiently transfected with purified EBV bacmid p2089 DNA for 48 h, and the cells were selected with 200 μg/ml hygromycin B for 2 weeks. All single clones were pooled and passaged to establish stable HNE-1–2089 cell lines. BHRF1 KO cells were generated by the CRISPR–Cas9 system. Briefly, stable HNE-1–2089 cells were infected with empty lentiviruses or lentiviruses expressing BHRF1 sgRNA for 48 h and then selected with puromycin (2 μg/ml) for 3 weeks. Single clones were picked, passed and screened by PCR, after which BHRF1 KO in the cells was validated by immunoblotting analysis with an anti-BHRF1 antibody.

### Plasmids

GFP-tagged wild-type and mutant BHRF1 constructs were amplified and subcloned into the pEGFP-C2 vector. GST-tagged BHRF1, wild-type AIM2 and AIM2 mutant constructs were subcloned into the pEBG vector. Flag-tagged vBcl2, M11, wild-type BHRF1 and BHRF1 mutant constructs were amplified and subcloned into the pCMV-3tag vector. Flag-AIM2, Flag-BHRF1 wild-type and the mutated constructs were subcloned into the pLVX-IRES-ZsGreen vector for lentiviral packaging.

The pro-IL-1β-DN-Gluc reporter, Flag-tagged NLRP1, NLRP3, NLRC4, AIM2, IFI16, RIG-I, ASC, pro-IL-1β, and pro-caspase-1 expression plasmids and the shRNA used for inflammasome-forming sensors were described previously [19], and all the sequences of shRNA were listed in S1 Table.

### Immunoprecipitation and western blotting analysis

In brief, one 10-cm dish of cells was transfected with 10–15 μg of plasmid for 48 h, after which the cells were collected and lysed in the presence of a protease inhibitor cocktail. For immunoprecipitation, the same amounts of cell lysates were precleaned and incubated with antibodies at 4 °C overnight and the complexes were precipitated with protein G-agarose (Sigma-Aldrich, 11243233001) or directly incubated with GST-affinity beads (Cytiva, 17075601) at 4 °C overnight. After washing five times, the immunoprecipitated complexes were subjected to SDS‒PAGE and immunoblotting analysis. For western blotting analysis, 40–60 μg/lane proteins from whole-cell extracts were separated via SDS–PAGE and subsequently transferred to NC or PVDF membranes. The membranes were blocked with 5% dry milk in PBS, incubated with primary antibodies at 4 °C overnight and subsequently incubated with species-matched IRDye 680- or IRDye 800-labeled secondary antibodies for 2 h at room temperature. After 5 washes with PBS containing 0.05% Tween-20, the images were visualized using a LI-COR Odyssey scanner. For protein concentration, the protein samples or the supernatants were precipitated with a final concentration of 10% (m/v) TCA at 4 °C. After washing two times with acetone and one time with methanol, the protein pellets were dissolved in SDS loading buffer and subjected to immunoblotting analysis.

### Luciferase assays

A Gaussia luciferase-based reporter for inflammasome activation was used to evaluate Caspase-1 activation and IL-1β cleavage. Briefly, the pro-IL-1β-DN-Gluc reporter was transfected into A549 cells with either an empty vector or shRNA and BHRF1-expressing plasmids in a 48-well plate for 36 h, after which the cells were infected with HSV-1 (MOI = 1) for 12 h. The supernatants were collected, and the secretion of Gaussia luciferase was measured using the reagent for Renilla luciferase activity and a TriStar multimode reader.

### Flow cytometry analysis

The mitochondrial number and membrane potential were detected using flow cytometry after staining with MitoTracker Red CMXRos (M7512, Thermo Fisher) and Rhodamine 123 (HY-D0816, MCE), respectively. Briefly, cells were seeded in 12 well plates, transfected with plasmids for 48 h, washed with PBS, and incubated with 100 μM MitoTracker or 10 µM Rhodamine 123 mitochondrial dye at 37 °C for 30 min in the dark. Then, the cells were collected, washed with PBS, and analyzed with a BD LSRFortessa FACS system (BD Biosciences).

### Detection of cell death

The pyroptosis was detected in living cells using PI staining. Briefly, the living cells were directly stained with PI solution, and then PI-positive cells were visualized using fluorescence microscope. The images of PI-positive cells were recorded and the percentages of pyroptotic cells were calculated in three independent experiments.

Cell cytotoxicity was determined using lactate dehydrogenase (LDH) release assay. Briefly, the supernatants of the cultured cells were collected and LDH release was measured using the LDH Cytotoxicity Assay Kit (Beyotime, C0016) according to the manufacturer’s manual. Three independent experiments were performed in triplicate and the relative levels of LDH release were calculated.

### Detection of EBV virion DNA and intracellular viral genomic DNA

To detect the virion production, P3HR1 cells were lytic induced by TPA plus NaB or BZLF1 overexpression for 96 h, or Akata+ cells were induced by IgG treatment for 96 h, and then the supernatants were collected and centrifuged twice at 12000 g for 10 min to remove the cell debris, after which the supernatants were digested with DNaseI at 37°C for 1 h, stopped by EDTA plus SDS, followed by proteinase K digestion at 55°C for 30 min. Finally, virion DNA was extracted using a standard phenol-chloroform procedure and determined by real-time PCR using the 2x SYBR Green (Accurate Biotechnology, AG11701).

For viral genomic DNA detection, the cells were collected after lytic induction for 48 h, and then total DNA was extracted by a GFamp Genomic DNA Kit (Genefist, GF2304) following the manufacturer’s manual. The viral genomic DNA in cells was analyzed by real-time PCR and normalized to β-actin genomic DNA. The primer pairs used are as follows:

EBNA1-F: 5’-CATTGAGTCGTCTCCCCTTTGGAAT-3’;
EBNA1-R: 5’-TCATAACAAGGTCCTTAATCGCATC-3’;
Actin-F: 5’-CACCATTGGCAATGAGCGGTTC-3’;
Actin-R: 5’-AGGTCTTTGCGGATGTCCACGT-3’.

### Immunofluorescence staining

The cells were fixed with 4% formaldehyde for 10 min, permeabilized with 0.1% Triton X-100 for 10 min, and blocked with 1% bovine serum albumin (BSA) in PBS for 1 h. Then, the cells were incubated with primary antibody at 4 °C overnight. After three washes with PBS containing 0.1% Triton X-100 and 0.1% BSA, the cells were incubated with Alexa Fluor-labeled anti-mouse or rabbit IgG secondary antibodies (Invitrogen) for 1 hour. The cells were counterstained with 4,6-diamidino-2-phenylindole (DAPI; Sigma-Aldrich) followed by three additional washes. The cells were mounted in antifade agent on glass slides and visualized with a confocal fluorescence microscope (LSM780, Zeiss).

### ASC oligomerization assay

After transfection with the ASC-expressing plasmid, the cells were collected, the cell lysates were centrifuged, and the supernatants were subsequently transferred to fresh tube. Equal amounts of total proteins were cross-linked with DSS (Sigma-Aldrich, S1885) at a final concentration of 2 mM at room temperature for 30 min, and the reaction was stopped by quenching buffer at a final concentration of 50 mM Tris–HCl (pH 7.5) at room temperature for 15 min. Finally, the protein samples were separated by SDS‒PAGE, and ASC oligomerization was detected by western blotting analysis with anti-ASC antibody.

### Caspase1 activity analysis

Briefly, the cells were collected and the whole-cell lysates were prepared. After the protein concentrations were measured, the activity of Caspase1 was analyzed by Caspase 1 Activity Assay Kit (Elabscience, E-CK-A381) following the manufacturer’s manual.

### Mitochondrial and cytoplasmic isolation

Briefly, the cells were collected, washed with ice-cold PBS, incubated with mitochondria isolation reagent (10 mM Tris–HCl (pH 7.5),10 mM NaCl,175 mM sucrose and 12.5 mM EDTA) for 30 min on ice and homogenized with 1 ml syringe. The cells and debris were pelleted by centrifugation at 1000x g for 10 min at 4 °C. The supernatant was further centrifuged at 10000g for 15 min at 4 °C. The supernatant (cytosolic fraction) and the pellet (mitochondrial fraction) were collected and analyzed by western blotting analysis.

### Detection of mtDNA release

Briefly, the mitochondria were removed and cytosolic fractions were prepared as described above, total DNA in cytoplasmic fractions and whole cells were extracted by a GFamp Genomic DNA Kit (Genefist, GF2304) according to the standard protocols. The amounts of mtDNA release were quantified by real-time PCR with SYBR Green, the numbers of mitochondrial ND1 gene (mtND1) in cytoplasmic fraction was normalized to nuclear beta-2 microglobulin (β2M). The primer pairs are used as follows:

ND1-F:5’-CACTTTCCACACAGACATCA-3’
ND1-R:5’- TGGTTAGGCTGGTGTTAGGG-3’
β2M -F:5’- TGTTCCTGCTGGGTAGCTCT -3’
β2M -R:5’- CCTCCATGATGCTGCTTACA -3’

### DNA–protein interactions in vitro and in cellular fractions

To detect AIM2-DNA binding in vitro, GST-AIM2 and GFP-BHRF1-His proteins were purified with GST-affinity beads and His-affinity beads, respectively. Then, the purified proteins were incubated with dsDNA overnight at 4 °C and pulled down with GST-affinity beads. To detect AIM2-dsDNA interaction in cells, the cytoplasmic fractions were isolated and then incubated anti-AIM2 antibody overnight at 4 °C, the complexes were precipitated with protein G-agarose. The DNA‒protein complexes were treated with 0.2 M NaCl for 1 h at 65 °C, followed by treatment with RNase A for 1 h, 0.5 M EDTA, 1 M Tris-HCl (pH 6.5) and 10 mg/ml Proteinase K for an additional 4 h. Finally, DNA was extracted using DNA gel extraction kit (Omega) according to the manufacturer’s instructions and detected by real-time PCR.

### CDOCKER molecular docking analysis

The sequences of the BHRF1 and AIM2-HIN domains were downloaded from NCBI, and their structures were downloaded from I-TASSER (http://zhanglab.ccmb.med.umich.edu/ITASSER/download/) and the Protein Data Bank (https://www.rcsb.org/), respectively. For the molecular docking, the structure of AIM2-HIIN was input into ZDOCK-server (https://zdock.umassmed.edu/) as a receptor, and the structure of BHRF1 was input as a ligand to generate the mode of 3D ligand–receptor interaction. Discovery Studio software CDOCKER (Accelrys Software, Inc., San Diego, CA, USA) was used to optimize and generate the best binding mode of 3D ligand–receptor interaction.

### Statistical analyses

All the statistical analyses were performed with Prism 8 software (GraphPad Software, La Jolla, CA, USA). One-way ANOVA with Sidak’s adjustment or Tukey’s multiple comparison test, and an unpaired t-test were used for two comparisons. A p value<0.05 indicated statistical significance.

## Supporting information

S1 Fig The inflammasome is activated during late EBV lytic replication. A. EBV-positive P3HR1 cells were treated with TPA plus NaB and then collected at the indicated time points. Whole cell extracts were analyzed by western blotting analysis for inflammasome activation. B. EBV-negative BJAB cells were treated or untreated with TPA plus NaB for 48 h or infected with HSV-1 (MOI = 1) for 12 h, then the whole cell extracts were analyzed by western blots as indicated. C. The pro-IL-1β-DN reporter was transfected into A549 cells with empty vector or one of EBV lytic ORF expressing plasmids. After cells were infected with HSV-1 (MOI = 1) for 12 h, the supernatants were collected and measured using a reagent for Renilla luciferase activity. The relative levels of triplicate analyses from three independent experiments were calculated and shown as the mean ± standard deviation. *, p < 0.01. Tukey’s multiple comparison test. D. THP-1 cells were infected with HSV-1 (MOI = 1) for different time, and then cells were collected and whole cell extracts were prepared and analyzed by western blots as indicated to detect the inflammasome activation. E. Supplementary images to Fig 1E. F-G. EBV-positive Akata+ cells were infected with empty and BHRF1-expressing lentiviruses (F), or scramble shRNA and two different shBHRF1 expressing lentiviruses (G) for 24 h, and then left untreated or treated with IgG for 48 h. The cells were collected and lysed, the cell extracts were subjected to western blotting analysis as indicated.
(TIF)

S2 Fig Identification of BHRF1-induced AIM2 inflammasome activation. A. The Gaussia luciferase-fused and 31 aa N-terminal truncated pro-IL-1β expression plasmid was co-transfected into A549 cells with the BHRF1 expression plasmid plus empty vector or shRNA as indicated for 24 h. Then, the cells were infected with HSV-1 (MOI = 1) for 12 h, after which the supernatants were collected, the Renilla luciferase activity was measured. The results are shown as the mean ± SD (n = 2). Sidak’s multiple comparisons test. *, p < 0.05. B. THP-1 cells were infected with empty or shAIM2-expressing lentiviruses plus empty or BHRF1-expressing lentiviruses, primed with 40 ng/ml TPA overnight, left uninfected or infected with HSV-1 (MOI = 1) for 12 h. The cell extracts were analyzed by western blotting analysis. C. P3HR1 cells were infected with empty or shAIM2-expressing lentiviruses. Twenty-four hours later, the cells were left untreated or treated with TPA and NaB for 48 h. Cells were collected, and the cell extracts were subjected to western blotting analysis as indicated.
(TIF)

S3 Fig Supplementary figures to Fig 3. A. P3HR1 cells were left untreated or treated with TPA and NaB for 48 h, then the cells were fixed and stained with anti-BHRF1, anti-AIM2 antibody and a species-matched AlexaFluor secondary antibody. Representative images were visualized by confocal microscopy and are shown. The coefficient of colocalization was determined by qualitative analysis of the fluorescence intensity of the selected areas. Scale bar: 5 μm. B. HNE-1–2089 cells were left untreated or treated with TPA and NaB for 36 h, then the cells were fixed and stained with anti-BHRF1, anti-ASC antibody and a species-matched AlexaFluor secondary antibody. Representative images were visualized by confocal microscopy and are shown. The coefficient of colocalization was determined by qualitative analysis of the fluorescence intensity of the selected areas. Scale bar: 5 μm.
(TIF)

S4 Fig Mapping the key regions for AIM2-BHRF1 interaction and inflammasome activation. A. Schematic diagrams showing the functional regions of AIM2 and the truncated mutants. B. Mapping the BHRF1-interacting region of AIM2. HEK293T cells were co-transfected with GST-tagged AIM2 full-length or truncated constructs and the Flag-BHRF1 expressing plasmid for 48 h, and then the cell extracts were subjected to immunoprecipitation and western blotting analysis as indicated. C. Schematic diagrams of BHRF1 wild-type and the deleted mutants. D. Mapping the AIM2-binding region in BHRF1. GFP-BHRF1 wild-type or deleted constructs were co-transfected into HEK293T cells with the GST-AIM2 expression plasmid for 36 h, after which the cell lysates were immunoprecipitated with GST affinity beads, and then whole-cell lysates and immunoprecipitated complexes were analyzed by western blotting analysis. E. HEK293T-CIA cells were transfected with GFP-BHRF1 WT or deleted constructs in the presence of AIM2-expressing plasmids for 36 h, and the cell extracts were analyzed for inflammasome activation. F. Empty vector, GFP-BHRF1 wild-type, CTD or aa109–191-expressing plasmid was co-transfected into HEK293T cells with GST-AIM2-expressing plasmid for 36 h. The cells were collected, and whole-cell lysates were immunoprecipitated with GST-affinity beads, the samples were subsequently analyzed by western blotting analysis with the indicated antibodies. G. Empty vector, GFP-BHRF1 wild-type, CTD or aa109–191-expressing plasmid was transfected into HEK293T-CIA cells in the presence of AIM2-expressing plasmids for 36 h, and the cell lysates were analyzed by western blotting analysis as indicated for inflammasome activation.
(TIF)

S5 Fig BHRF1-mediated AIM2 inflammasome activation is independent of its anti-apoptotic and autophagic function. A. The structure of BHRF1-AIM2 HIN-dsDNA complexes in different horizontal and vertical angles. B. GST-fused or Flag-tagged AIM2 WT or E186A expressing plasmids were transfected alone or together into HEK293T cells for 36 h and then infected with HSV-1 (MOI = 1) for 12 h. The cell lysates were prepared and subjected to immunoprecipitation and western blotting analysis as indicated to detect AIM2-AIM2 self-interaction. C. Empty vector, AIM2 WT, E186A or N241A expressing plasmid was transfected into HEK293T-CIA cells for 24h, and then cells were infected with HSV-1 (MOI = 1) for 12 h. Cell lysates were prepared and analyzed by western blots as indicated to detect the inflammasome activation. D-E. BHRF1 KO HNE-1–2089 cells were transfected with empty vector, BHRF1 wild-type, R162A or F164A constructs for 24 h, induced with TPA plus NaB for 48 h and then treated with 20 µM cisplatin for 24 h to induce apoptosis (D), or left untreated or treated with HBSS as an inducer of autophagy (E). Whole-cell extracts were analyzed by western blotting analysis as indicated. F. BHRF1 KO HNE-1–2089 cells were transfected with empty vector, BHRF1 wild-type, G99A or R100D constructs for 24 h, induced with TPA plus NaB for 48 h. Whole-cell extracts were prepared and analyzed by western blotting analysis to detect the inflammasome activation.
(TIF)

S6 Fig BHRF1 enhances AIM2-ASC recruitment and AIM2-dsDNA binding. A. Empty or GFP-BHRF1-expressing plasmid was co-transfected into HEK293T cells with GST-AIM2 and Flag-ASC expressing plasmids for 24 h, then the cells were left untreated or treated with 5 μg/mL poly(dA:dT) for 16 h. Cell lysates were immunoprecipitated with GST-affinity beads, and the samples were detected by western blotting analysis to detect AIM2-ASC recruitment. B. The purified GST-AIM2, Flag-AIM2-His and GFP-BHRF1-His proteins were mixed and incubated overnight at 4 °C in the absence or presence of purified dsDNA, after which the mixtures were pulled down with GST-affinity beads and then subsequently analyzed by western blotting analysis as indicated to detect in vitro AIM2-AIM2 polymerization.
(TIF)

S7 Fig BHRF1and AIM2 expression promotes EBV virion production. A. The relative levels of viral gene expression in Fig 5A were detected by real-time PCR and shown. The results are show as the mean ± SD (n = 3). Tukey’s multiple comparisons test. ****, p < 0.0001. B-C. P3HR1 cells were infected with empty, BHRF1 WT, G99A or R100D expressing lentiviruses for 24 h and then left untreated or treated with TPA plus NaB. After 48h, the cell extracts were prepared and analyzed by western blotting analysis (B). After 96 h, the extracellular virion DNA was analyzed by real-time PCR (C). Data are shown as the mean ± SD (n = 3), Tukey’s multiple comparisons test. ***, p < 0.0005. D-G. Akata+ cells were infected with empty, BHRF1 WT, R162A, F164A-expressing lentiviruses (D) or scramble shRNA or shBHRF1-expressing lentiviruses (E) for 24 h and then left untreated or treated with IgG. The levels of gene expression were analyzed by western blotting analysis (D-E) and the extracellular virion DNA was analyzed by real-time PCR (F-G) as described above. The data are shown as the mean ± SD (n = 3). Tukey’s multiple comparisons test. ****, p < 0.0001. H-I. P3HR1 cells were infected with empty, BHRF1 WT, R162A, F164A-expressing lentiviruses (H) or scramble shRNA or shBHRF1-expressing lentiviruses (I) for 24 h, and then infected with BZLF1-expressing lentiviruses to induce lytic replication. Ninety-six hours later, the extracellular virion DNA was analyzed by real-time PCR. The results are show as the mean ± SD (n = 3). Tukey’s multiple comparisons test. ****, p < 0.0001.
(TIF)

S8 Fig AIM2 expression promotes EBV virion production. A. The relative levels of viral gene expression in Fig 5E were detected by real-time PCR and shown. The results are show as the mean ± SD (n = 3). Tukey’s multiple comparisons test. ****, p < 0.0001. B-C. P3HR1 cells were infected with empty, AIM2-expressing (B) or shAIM2-expressing(C) lentiviruses for 24 h, after which the cells were treated with TPA plus NaB for lytic induction. Total DNA was extracted after 48 h of induction, and the late lytic gene expression was detected by real-time PCR. The results are show as the mean ± SD (n = 3). Tukey’s multiple comparisons test. **, p < 0.05; ***, P < 0.005; ****, P < 0.0001. D-E. P3HR1 cells were infected with different amounts of empty or AIM2-expressing lentiviruses for 24 h, after which the cells were treated with TPA plus NaB for lytic induction. Extracellular virions in the supernatants were collected after 96 h of induction, and the virion DNA was extracted and analyzed by real-time PCR (C). Total DNA was extracted after 48 h of induction, and the amount of viral genomic DNA was quantified by real-time PCR and normalized to the amount of cellular genomic actin (D). The virion production and viral DNA replication curves were generated and are shown as mean ± SD (n = 3), and the level of AIM2 expression was detected by western blots. E-F. P3HR1 cells were infected with empty or shAIM2-expressing lentiviruses for 24 h and then left untreated or treated with TPA plus NaB. After 72 h treatment, the cells were collected and the cell extracts were analyzed by western blotting analysis (E). Extracellular virions were collected after 96 h induction, and virion DNA was extracted and analyzed by real-time PCR (F). The results are shown as the mean ± SD (n = 3), Tukey’s multiple comparisons test. ****, p < 0.0001.
(TIF)

S9 Fig BHRF1-induced AIM2 inflammasome activation does not induce mitochondrial dysfunction, apoptosis or necroptosis. A-B. BHRF1 WT and KO HNE-1–2089 cells were infected with empty, BHRF1 wild-type or mutant‐expressing lentiviruses for 24 h, then induced with TPA plus NaB for 48 h and stained with PI dye. Images of PI-positive cells and total cells were acquired by inverted fluorescence microscopy. Representative images of pyroptotic cells are shown (A) and the percentages of pyroptotic cells were calculated from six random fields in two independent experiments (B). Scale bar: 15 μm. C. The cells were infected with lentiviruses and induced as described above. After 48 h of induction, the mtDNA of cytoplasmic fraction was extracted and analyzed by real-time PCR. The results are shown as the mean ± SD (n = 3). Tukey’s multiple comparisons test, ****, p < 0.0001. D-E. BHRF1 WT and KO HNE-1–2089 cells were infected with empty, BHRF1 wild-type or mutant-expressing lentiviruses and induced as described above. The cells were stained with MitoTracker Red CMXRos (D) and Rhodamine 123 (E). The results were subsequently analyzed by fluorescent flow cytometry, and representative images are shown. The mean fluorescence intensity was also analyzed in two independent experiments and shown. Tukey’s multiple comparisons test, ***, p < 0.0005; ****, p < 0.0001. F-H. BHRF1 KO HNE-1–2089 cells were infected with empty, BHRF1 wild-type or mutant‐expressing lentiviruses for 24 h and then were induced with TPA plus NaB for 48 h. The cells were collected, and the cell extracts were analyzed as indicated to detect the cleavage of caspase-3 (F), or to detect RIPK3 phosphorylation with Sucrose treatment (500 mM) for 90min as a positive control (G). The cells were left untreated or treated with 20 µM cisplatin for 24 h to induce cell death, and then the supernatants were collected and the relative levels of LDH release were measured and shown (H). The results are shown as the mean ± SD (n = 3). ****, p < 0.0001 by Tukey’s multiple comparisons test.
(TIF)

S10 Fig KAP1 and GSDMD expression are required for EBV lytic replication. A-D. P3HR1 cells were infected with empty or shGSDMD (A-B) or shKAP1 (C-D) expressing lentiviruses for 24 h and then left untreated or treated with TPA plus NaB. The cells were collected after 48h, and the cell extracts were analyzed by western blotting analysis as indicated (A, C). Extracellular virions were collected after 96 h induction, and virion DNA was extracted and analyzed by real-time PCR (B, D). The results are shown as the mean ± SD (n = 3), ****, p < 0.0001 by Tukey’s multiple comparisons test.
(TIF)

S1 Table The sequences of shRNA. All the shRNA used in manuscript are show in this table.
(PDF)
