# Supplementary figures and images for: The viral BCL2 protein BHRF1 of Epstein–Barr virus promotes AIM2 inflammasome activation to facilitate lytic replication
Source: PLoS Pathog. 2025 Sep 22;21(9):e1013509. doi: 10.1371/journal.ppat.1013509 (PMC12483278; doi:10.1371/journal.ppat.1013509)

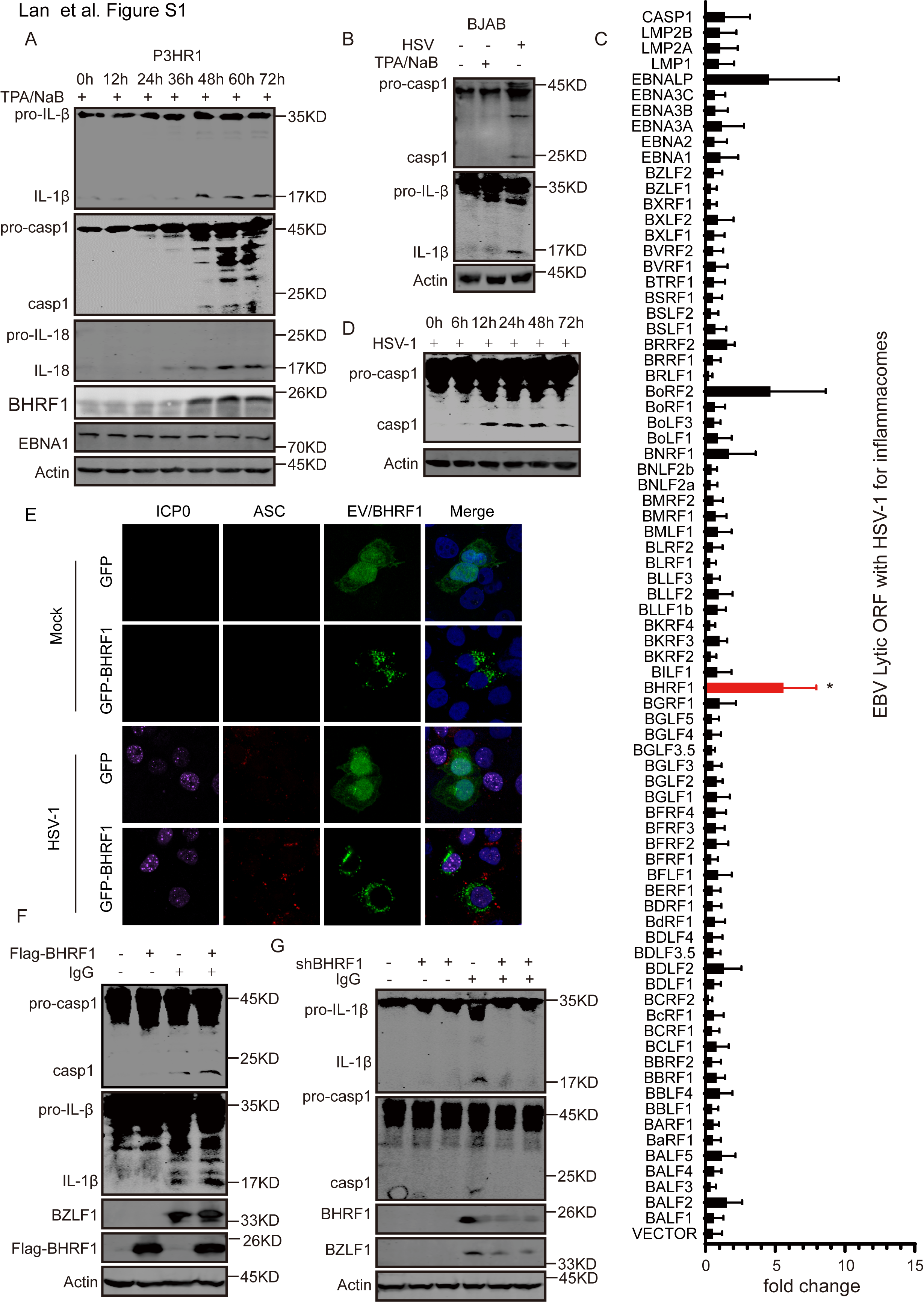

Supplement: S1 Fig — A. EBV-positive P3HR1 cells were treated with TPA plus NaB and then collected at the indicated time points. Whole cell extracts were analyzed by western blotting analysis for inflammasome activation. B. EBV-negative BJAB cells were treated or untreated with TPA plus NaB for 48 h or infected with HSV-1 (MOI = 1) for 12 h, then the whole cell extracts were analyzed by western blots as indicated. C. The pro-IL-1β-DN reporter was transfected into A549 cells with empty vector or one of EBV lytic ORF expressing plasmids. After cells were infected with HSV-1 (MOI = 1) for 12 h, the supernatants were collected and measured using a reagent for Renilla luciferase activity. The relative levels of triplicate analyses from three independent experiments were calculated and shown as the mean ± standard deviation. *, p < 0.01. Tukey’s multiple comparison test. D. THP-1 cells were infected with HSV-1 (MOI = 1) for different time, and then cells were collected and whole cell extracts were prepared and analyzed by western blots as indicated to detect the inflammasome activation. E. Supplementary images to Fig 1E. F-G. EBV-positive Akata+ cells were infected with empty and BHRF1-expressing lentiviruses (F), or scramble shRNA and two different shBHRF1 expressing lentiviruses (G) for 24 h, and then left untreated or treated with IgG for 48 h. The cells were collected and lysed, the cell extracts were subjected to western blotting analysis as indicated. (TIF) [file ppat.1013509.s001.tif]

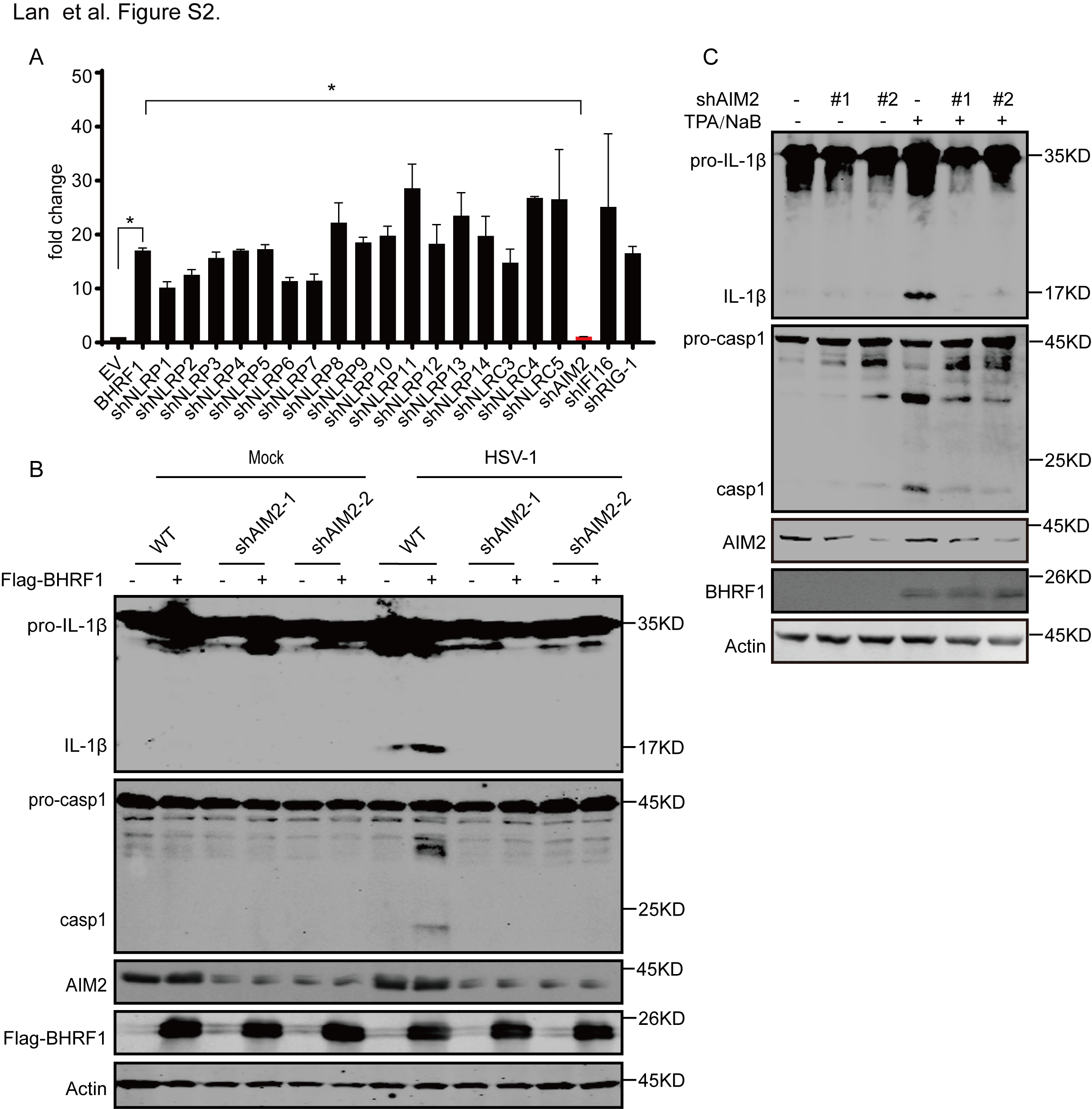

Supplement: S2 Fig — A. The Gaussia luciferase-fused and 31 aa N-terminal truncated pro-IL-1β expression plasmid was co-transfected into A549 cells with the BHRF1 expression plasmid plus empty vector or shRNA as indicated for 24 h. Then, the cells were infected with HSV-1 (MOI = 1) for 12 h, after which the supernatants were collected, the Renilla luciferase activity was measured. The results are shown as the mean ± SD (n = 2). Sidak’s multiple comparisons test. *, p < 0.05. B. THP-1 cells were infected with empty or shAIM2-expressing lentiviruses plus empty or BHRF1-expressing lentiviruses, primed with 40 ng/ml TPA overnight, left uninfected or infected with HSV-1 (MOI = 1) for 12 h. The cell extracts were analyzed by western blotting analysis. C. P3HR1 cells were infected with empty or shAIM2-expressing lentiviruses. Twenty-four hours later, the cells were left untreated or treated with TPA and NaB for 48 h. Cells were collected, and the cell extracts were subjected to western blotting analysis as indicated. (TIF) [file ppat.1013509.s002.tif]

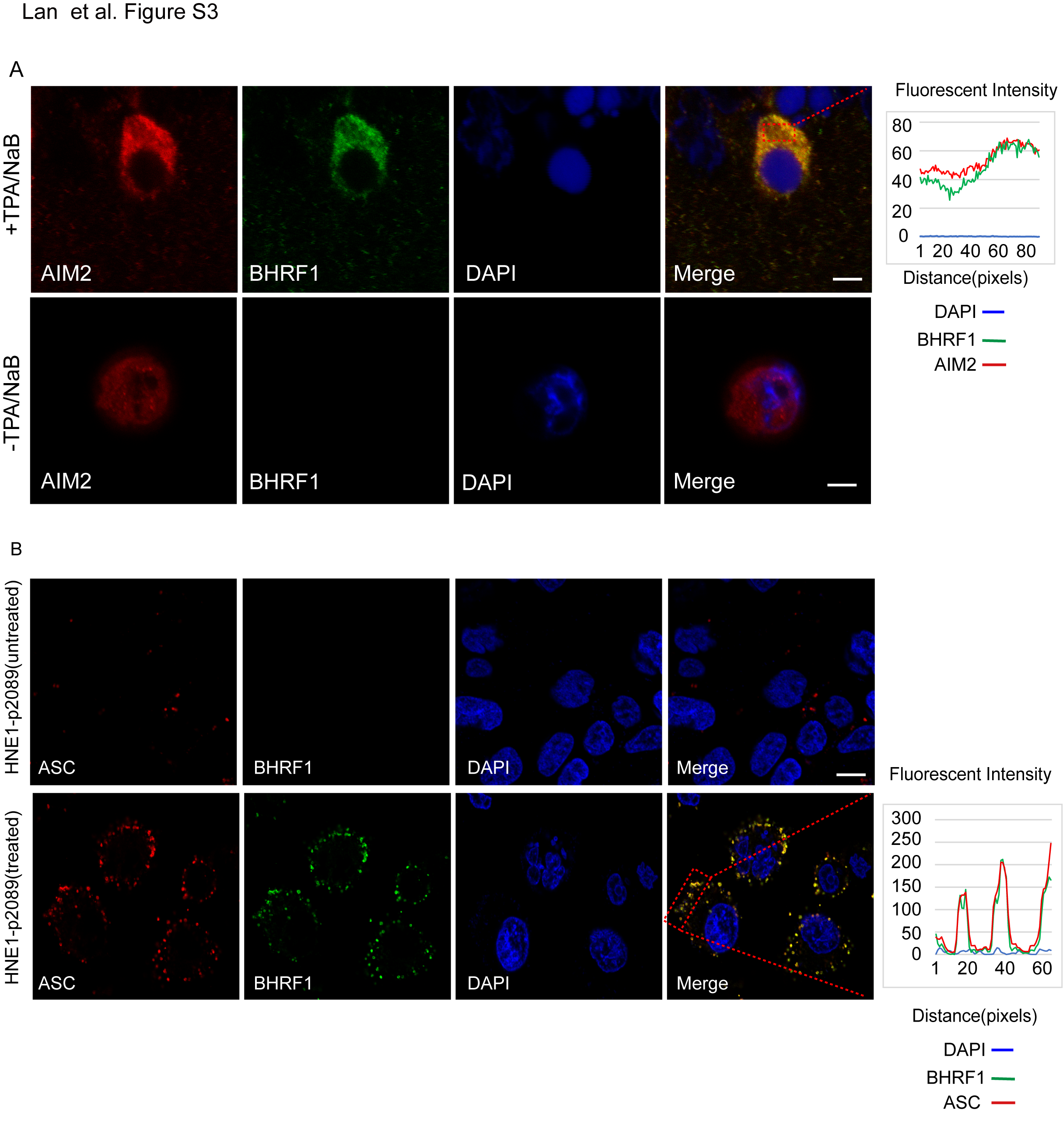

Supplement: S3 Fig — P3HR1 cells were left untreated or treated with TPA and NaB for 48 h, then the cells were fixed and stained with anti-BHRF1, anti-AIM2 antibody and a species-matched AlexaFluor secondary antibody. Representative images were visualized by confocal microscopy and are shown. The coefficient of colocalization was determined by qualitative analysis of the fluorescence intensity of the selected areas. Scale bar: 5 μm. B. HNE-1–2089 cells were left untreated or treated with TPA and NaB for 36 h, then the cells were fixed and stained with anti-BHRF1, anti-ASC antibody and a species-matched AlexaFluor secondary antibody. Representative images were visualized by confocal microscopy and are shown. The coefficient of colocalization was determined by qualitative analysis of the fluorescence intensity of the selected areas. Scale bar: 5 μm. (TIF) [file ppat.1013509.s003.tif]

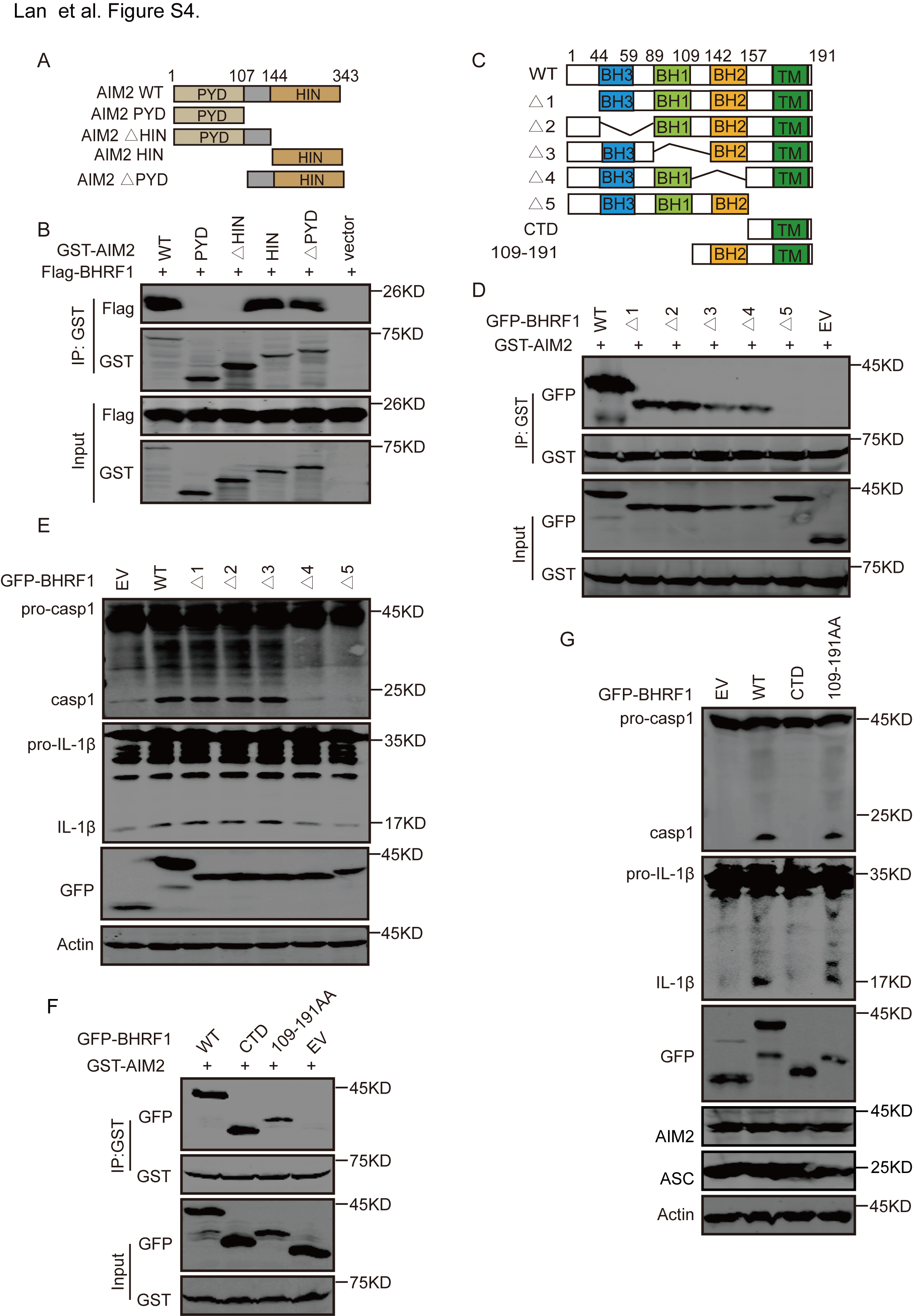

Supplement: S4 Fig — A. Schematic diagrams showing the functional regions of AIM2 and the truncated mutants. B. Mapping the BHRF1-interacting region of AIM2. HEK293T cells were co-transfected with GST-tagged AIM2 full-length or truncated constructs and the Flag-BHRF1 expressing plasmid for 48 h, and then the cell extracts were subjected to immunoprecipitation and western blotting analysis as indicated. C. Schematic diagrams of BHRF1 wild-type and the deleted mutants. D. Mapping the AIM2-binding region in BHRF1. GFP-BHRF1 wild-type or deleted constructs were co-transfected into HEK293T cells with the GST-AIM2 expression plasmid for 36 h, after which the cell lysates were immunoprecipitated with GST affinity beads, and then whole-cell lysates and immunoprecipitated complexes were analyzed by western blotting analysis. E. HEK293T-CIA cells were transfected with GFP-BHRF1 WT or deleted constructs in the presence of AIM2-expressing plasmids for 36 h, and the cell extracts were analyzed for inflammasome activation. F. Empty vector, GFP-BHRF1 wild-type, CTD or aa109–191-expressing plasmid was co-transfected into HEK293T cells with GST-AIM2-expressing plasmid for 36 h. The cells were collected, and whole-cell lysates were immunoprecipitated with GST-affinity beads, the samples were subsequently analyzed by western blotting analysis with the indicated antibodies. G. Empty vector, GFP-BHRF1 wild-type, CTD or aa109–191-expressing plasmid was transfected into HEK293T-CIA cells in the presence of AIM2-expressing plasmids for 36 h, and the cell lysates were analyzed by western blotting analysis as indicated for inflammasome activation. (TIF) [file ppat.1013509.s004.tif]

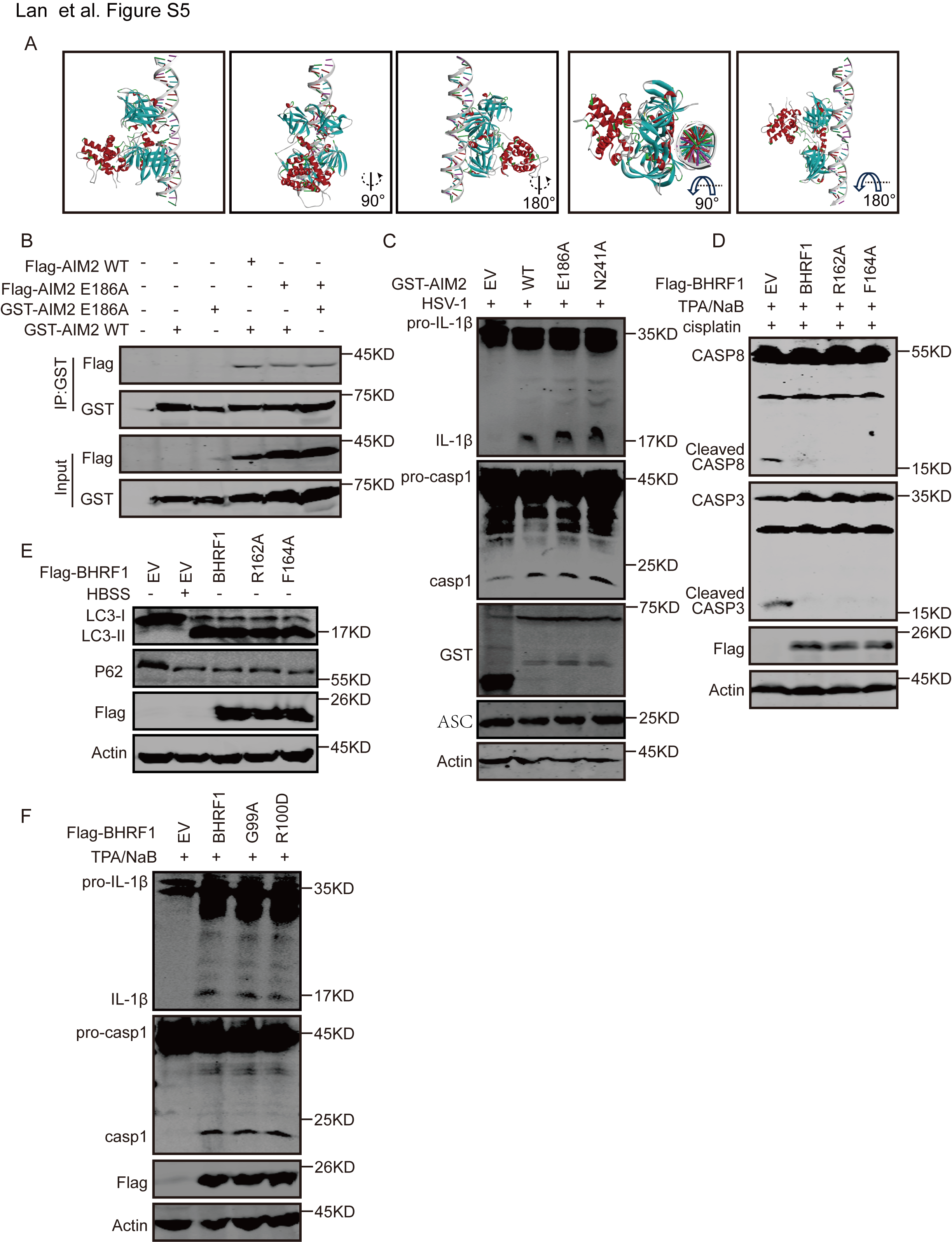

Supplement: S5 Fig — A. The structure of BHRF1-AIM2 HIN-dsDNA complexes in different horizontal and vertical angles. B. GST-fused or Flag-tagged AIM2 WT or E186A expressing plasmids were transfected alone or together into HEK293T cells for 36 h and then infected with HSV-1 (MOI = 1) for 12 h. The cell lysates were prepared and subjected to immunoprecipitation and western blotting analysis as indicated to detect AIM2-AIM2 self-interaction. C. Empty vector, AIM2 WT, E186A or N241A expressing plasmid was transfected into HEK293T-CIA cells for 24h, and then cells were infected with HSV-1 (MOI = 1) for 12 h. Cell lysates were prepared and analyzed by western blots as indicated to detect the inflammasome activation. D-E. BHRF1 KO HNE-1–2089 cells were transfected with empty vector, BHRF1 wild-type, R162A or F164A constructs for 24 h, induced with TPA plus NaB for 48 h and then treated with 20 µM cisplatin for 24 h to induce apoptosis (D), or left untreated or treated with HBSS as an inducer of autophagy (E). Whole-cell extracts were analyzed by western blotting analysis as indicated. F. BHRF1 KO HNE-1–2089 cells were transfected with empty vector, BHRF1 wild-type, G99A or R100D constructs for 24 h, induced with TPA plus NaB for 48 h. Whole-cell extracts were prepared and analyzed by western blotting analysis to detect the inflammasome activation. (TIF) [file ppat.1013509.s005.tif]

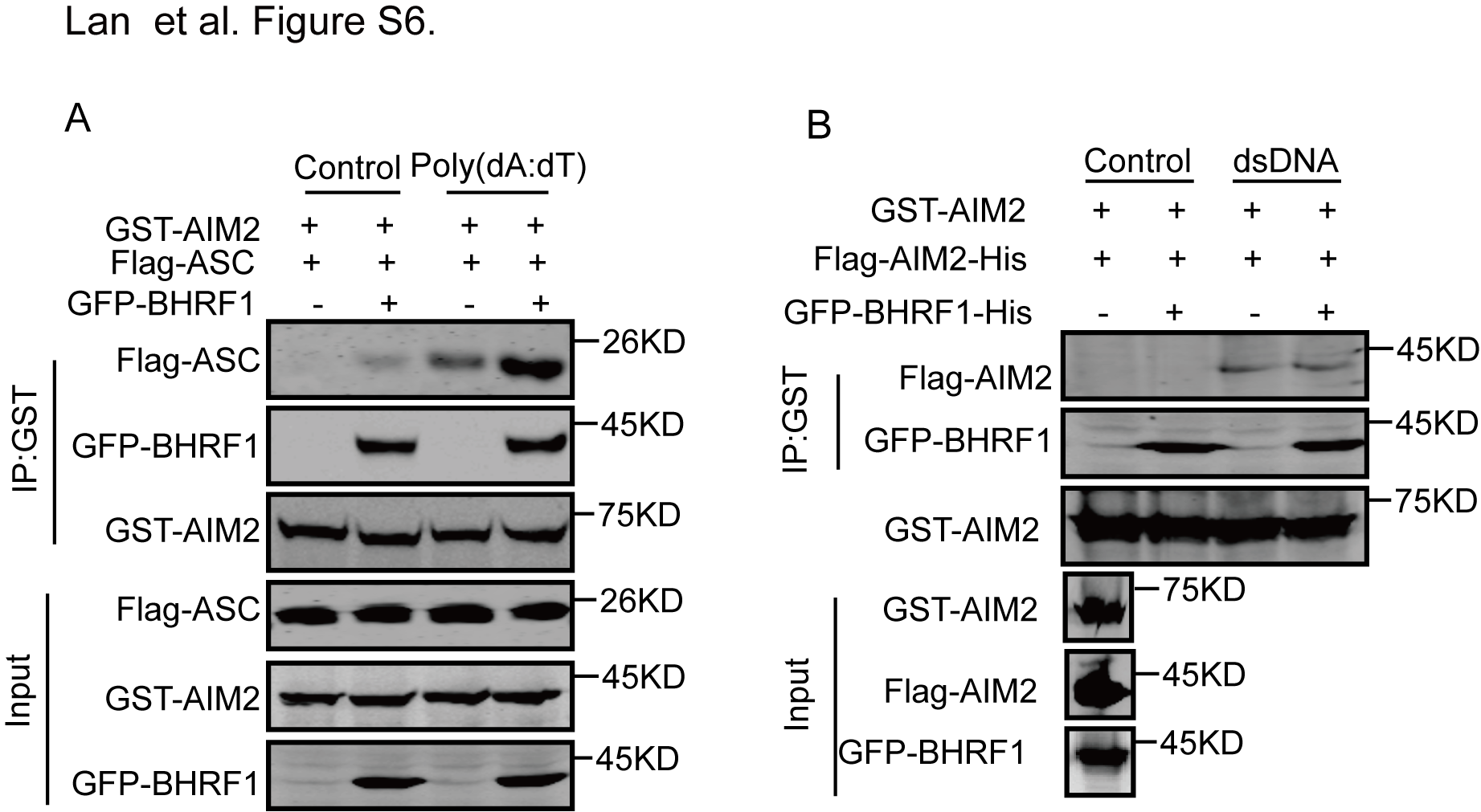

Supplement: S6 Fig — A. Empty or GFP-BHRF1-expressing plasmid was co-transfected into HEK293T cells with GST-AIM2 and Flag-ASC expressing plasmids for 24 h, then the cells were left untreated or treated with 5 μg/mL poly(dA:dT) for 16 h. Cell lysates were immunoprecipitated with GST-affinity beads, and the samples were detected by western blotting analysis to detect AIM2-ASC recruitment. B. The purified GST-AIM2, Flag-AIM2-His and GFP-BHRF1-His proteins were mixed and incubated overnight at 4 °C in the absence or presence of purified dsDNA, after which the mixtures were pulled down with GST-affinity beads and then subsequently analyzed by western blotting analysis as indicated to detect in vitro AIM2-AIM2 polymerization. (TIF) [file ppat.1013509.s006.tif]

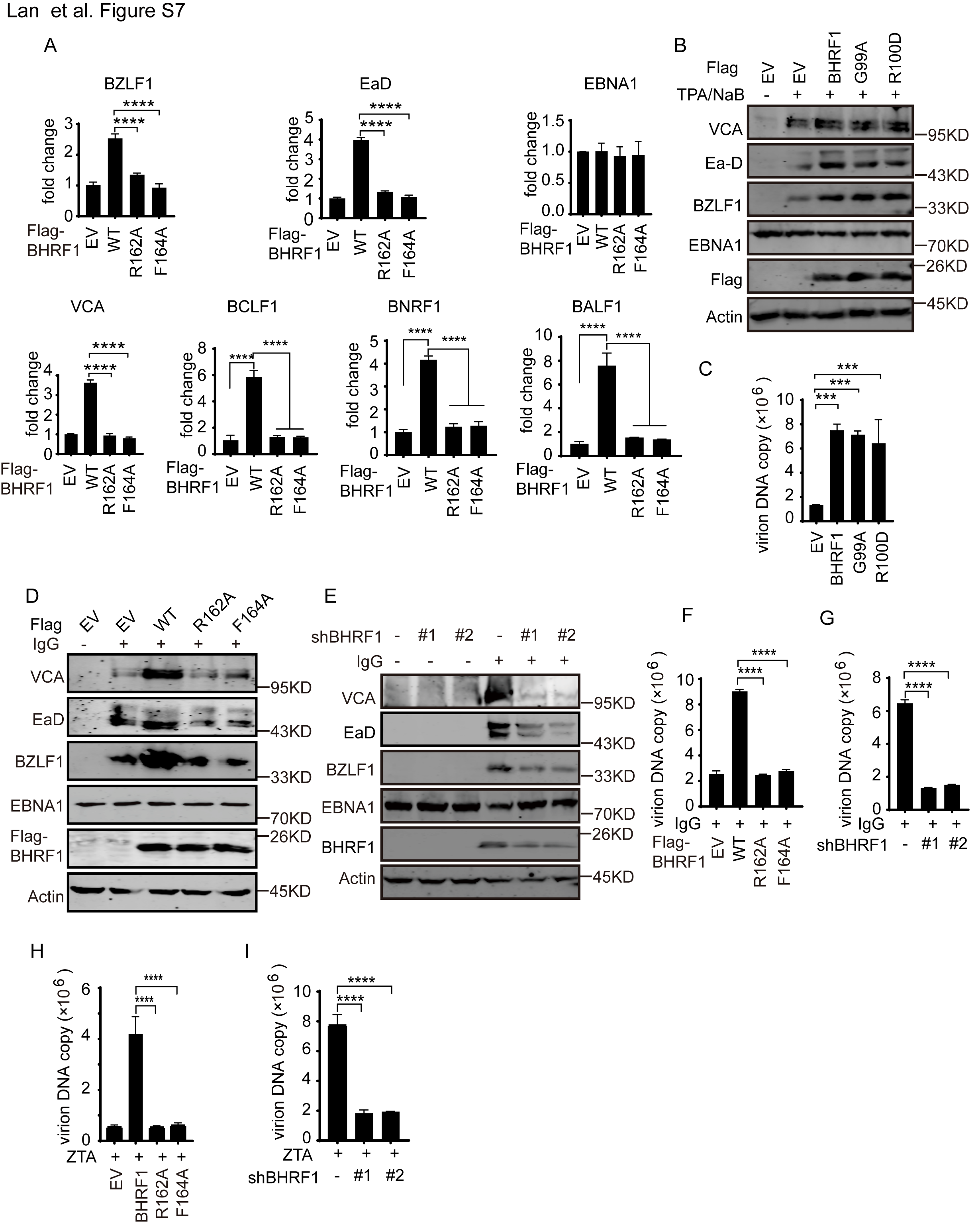

Supplement: S7 Fig — A. The relative levels of viral gene expression in Fig 5A were detected by real-time PCR and shown. The results are show as the mean ± SD (n = 3). Tukey’s multiple comparisons test. ****, p < 0.0001. B-C. P3HR1 cells were infected with empty, BHRF1 WT, G99A or R100D expressing lentiviruses for 24 h and then left untreated or treated with TPA plus NaB. After 48h, the cell extracts were prepared and analyzed by western blotting analysis (B). After 96 h, the extracellular virion DNA was analyzed by real-time PCR (C). Data are shown as the mean ± SD (n = 3), Tukey’s multiple comparisons test. ***, p < 0.0005. D-G. Akata+ cells were infected with empty, BHRF1 WT, R162A, F164A-expressing lentiviruses (D) or scramble shRNA or shBHRF1-expressing lentiviruses (E) for 24 h and then left untreated or treated with IgG. The levels of gene expression were analyzed by western blotting analysis (D-E) and the extracellular virion DNA was analyzed by real-time PCR (F-G) as described above. The data are shown as the mean ± SD (n = 3). Tukey’s multiple comparisons test. ****, p < 0.0001. H-I. P3HR1 cells were infected with empty, BHRF1 WT, R162A, F164A-expressing lentiviruses (H) or scramble shRNA or shBHRF1-expressing lentiviruses (I) for 24 h, and then infected with BZLF1-expressing lentiviruses to induce lytic replication. Ninety-six hours later, the extracellular virion DNA was analyzed by real-time PCR. The results are show as the mean ± SD (n = 3). Tukey’s multiple comparisons test. ****, p < 0.0001. (TIF) [file ppat.1013509.s007.tif]

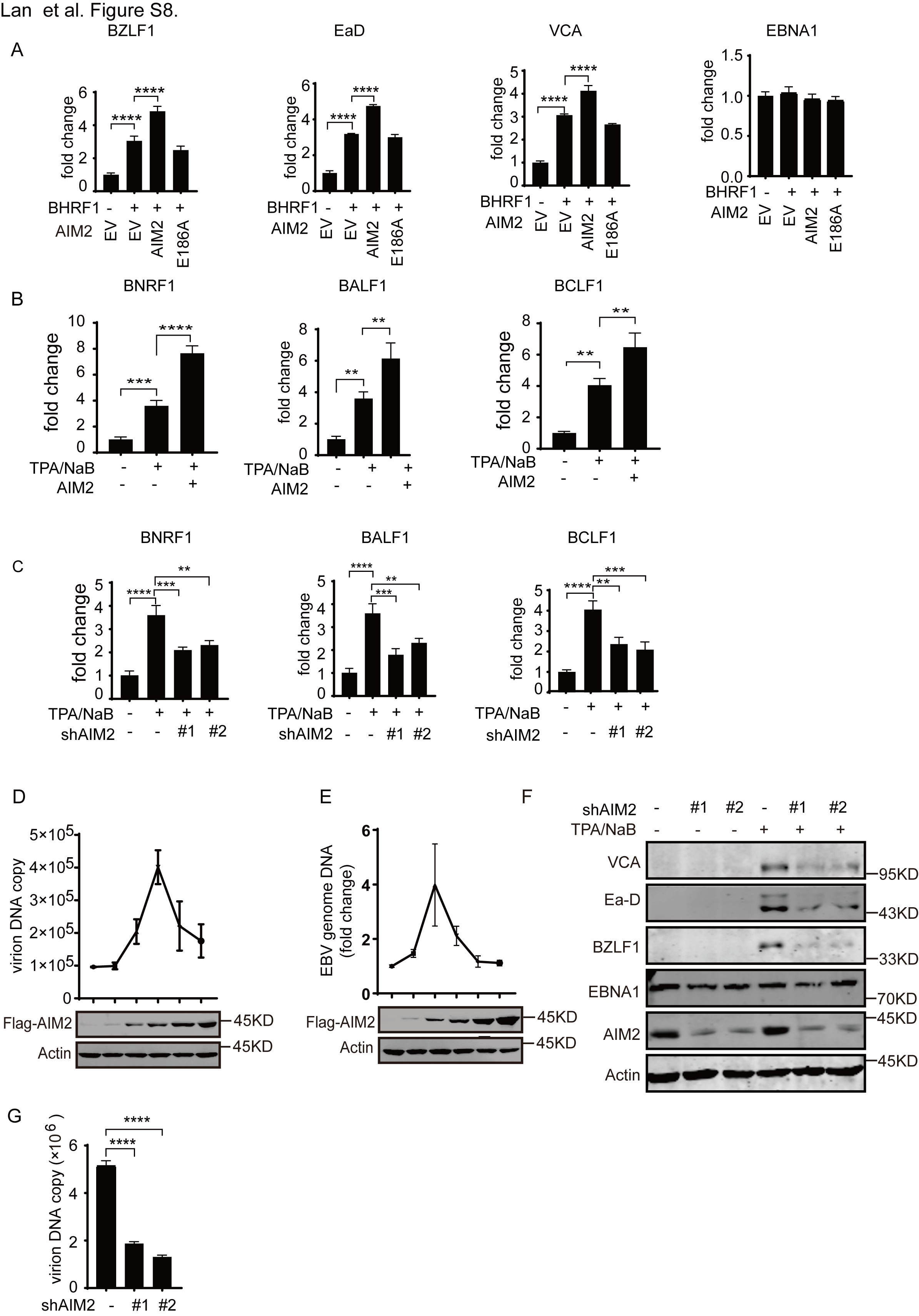

Supplement: S8 Fig — A. The relative levels of viral gene expression in Fig 5E were detected by real-time PCR and shown. The results are show as the mean ± SD (n = 3). Tukey’s multiple comparisons test. ****, p < 0.0001. B-C. P3HR1 cells were infected with empty, AIM2-expressing (B) or shAIM2-expressing(C) lentiviruses for 24 h, after which the cells were treated with TPA plus NaB for lytic induction. Total DNA was extracted after 48 h of induction, and the late lytic gene expression was detected by real-time PCR. The results are show as the mean ± SD (n = 3). Tukey’s multiple comparisons test. **, p < 0.05; ***, P < 0.005; ****, P < 0.0001. D-E. P3HR1 cells were infected with different amounts of empty or AIM2-expressing lentiviruses for 24 h, after which the cells were treated with TPA plus NaB for lytic induction. Extracellular virions in the supernatants were collected after 96 h of induction, and the virion DNA was extracted and analyzed by real-time PCR (C). Total DNA was extracted after 48 h of induction, and the amount of viral genomic DNA was quantified by real-time PCR and normalized to the amount of cellular genomic actin (D). The virion production and viral DNA replication curves were generated and are shown as mean ± SD (n = 3), and the level of AIM2 expression was detected by western blots. E-F. P3HR1 cells were infected with empty or shAIM2-expressing lentiviruses for 24 h and then left untreated or treated with TPA plus NaB. After 72 h treatment, the cells were collected and the cell extracts were analyzed by western blotting analysis (E). Extracellular virions were collected after 96 h induction, and virion DNA was extracted and analyzed by real-time PCR (F). The results are shown as the mean ± SD (n = 3), Tukey’s multiple comparisons test. ****, p < 0.0001. (TIF) [file ppat.1013509.s008.tif]

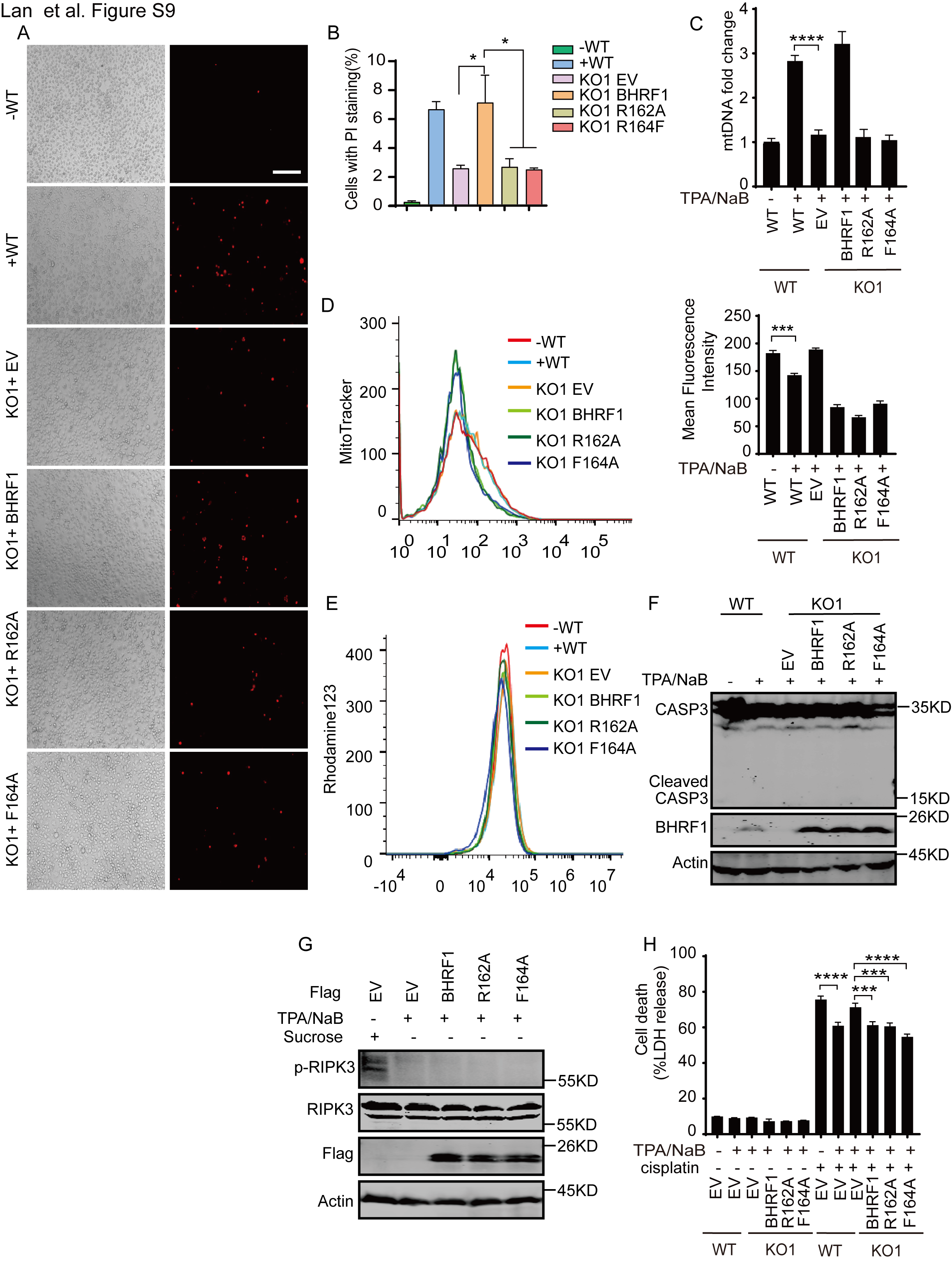

Supplement: S9 Fig — A-B. BHRF1 WT and KO HNE-1–2089 cells were infected with empty, BHRF1 wild-type or mutant‐expressing lentiviruses for 24 h, then induced with TPA plus NaB for 48 h and stained with PI dye. Images of PI-positive cells and total cells were acquired by inverted fluorescence microscopy. Representative images of pyroptotic cells are shown (A) and the percentages of pyroptotic cells were calculated from six random fields in two independent experiments (B). Scale bar: 15 μm. C. The cells were infected with lentiviruses and induced as described above. After 48 h of induction, the mtDNA of cytoplasmic fraction was extracted and analyzed by real-time PCR. The results are shown as the mean ± SD (n = 3). Tukey’s multiple comparisons test, ****, p < 0.0001. D-E. BHRF1 WT and KO HNE-1–2089 cells were infected with empty, BHRF1 wild-type or mutant-expressing lentiviruses and induced as described above. The cells were stained with MitoTracker Red CMXRos (D) and Rhodamine 123 (E). The results were subsequently analyzed by fluorescent flow cytometry, and representative images are shown. The mean fluorescence intensity was also analyzed in two independent experiments and shown. Tukey’s multiple comparisons test, ***, p < 0.0005; ****, p < 0.0001. F-H. BHRF1 KO HNE-1–2089 cells were infected with empty, BHRF1 wild-type or mutant‐expressing lentiviruses for 24 h and then were induced with TPA plus NaB for 48 h. The cells were collected, and the cell extracts were analyzed as indicated to detect the cleavage of caspase-3 (F), or to detect RIPK3 phosphorylation with Sucrose treatment (500 mM) for 90min as a positive control (G). The cells were left untreated or treated with 20 µM cisplatin for 24 h to induce cell death, and then the supernatants were collected and the relative levels of LDH release were measured and shown (H). The results are shown as the mean ± SD (n = 3). ****, p < 0.0001 by Tukey’s multiple comparisons test. (TIF) [file ppat.1013509.s009.tif]

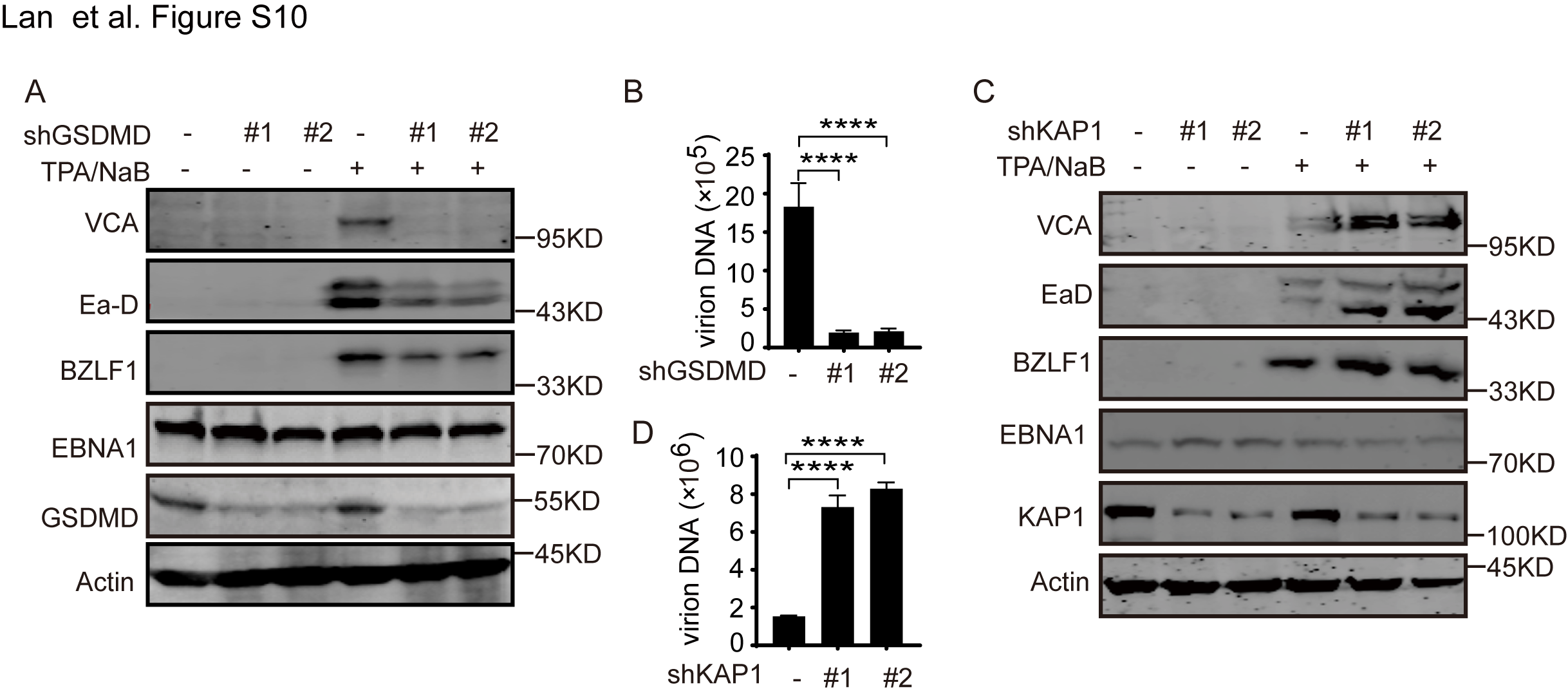

Supplement: S10 Fig — A-D. P3HR1 cells were infected with empty or shGSDMD (A-B) or shKAP1 (C-D) expressing lentiviruses for 24 h and then left untreated or treated with TPA plus NaB. The cells were collected after 48h, and the cell extracts were analyzed by western blotting analysis as indicated (A, C). Extracellular virions were collected after 96 h induction, and virion DNA was extracted and analyzed by real-time PCR (B, D). The results are shown as the mean ± SD (n = 3), ****, p < 0.0001 by Tukey’s multiple comparisons test. (TIF) [file ppat.1013509.s010.tif]
