# Supplementary material for: The viral BCL2 protein BHRF1 of Epstein–Barr virus promotes AIM2 inflammasome activation to facilitate lytic replication
Source: PLoS Pathog. 2025 Sep 22;21(9):e1013509. doi: 10.1371/journal.ppat.1013509 (PMC12483278; doi:10.1371/journal.ppat.1013509)
Supplement: S1 Table — All the shRNA used in manuscript are show in this table. (PDF) [file ppat.1013509.s011.pdf]

Table S1. The sequences of shRNA

Oligonucleotides	Sequences
shRIG-I	CCGGAGCACTTGTGGACGCTTTAAACTCGAGTTTAAAGCGTCCACAAGTGCTTTTTG
shIFI16	CCGGCTGGATGTCATTGACGATAATCTCGAGATTATCGTCAATGACATCCAGTTTTTG
shNLRP1	CCGGCGTTTCCTATTGGGCCTGTTACTCGAGTAACAGGCCCAATAGGAAACGTTTTTG
shNLRP2	CCGGGCTGAATCACATAGGAGTTAACTCGAGTTAACTCCTATGTGATTCAGCTTTTTG
shNLRP3	CCGGGTGGATCTAGCCACGCTAATGCTCGAGCATTAGCGTGGCTAGATCCACTTTTTG
shNLRP4	CCGGCAAATGACTTTGCAGCTTGAACTCGAGTTCAAGCTGCAAAGTCATTTGTTTTTG
shNLRP5	CCGGCCGTAACCTAAGATCCCTCAACTCGAGTTGAGGGATCTTAGGTTACGGTTTTTG
shNLRP6	CCGGCCAAAGTGCAGACGCTGTTTCCTCGAGGAAACAGCGTCTGCACTTTGGTTTTTG
shNLRP7	CCGGGCGACTGTGAACATCTTGGAACTCGAGTTCCAAGATGTTCACAGTCGCTTTTTG
shNLRP8	CCGGCAAGGTGTCTTTCGGTAATAACTCGAGTTATTACCGAAAGACACCTTGTTTTTG
shNLRP9	CCGGCCCTGAAACTTGGGCATAATGCTCGAGCATTATGCCCAAGTTTCAGGGTTTTTG
shNLRP10	CCGGCTCTACAAAGCGTGTCAGGTTCTCGAGAACCTGACACGCTTTGTAGAGTTTTTG
shNLRP11	CCGGCCGTTACAAGTTCATACACTTCTCGAGAAGTGTATGAACTTGTAACGGTTTTTG
shNLRP12	CCGGCGACCTTTACCTGACCAACAACTCGAGTTGTTGGTCAGGTAAAGGTCGTTTTTG
shNLRP13	CCGGCGTATCTTTGAAGTTGACCTTCTCGAGAAGGTCAACTTCAAAGATACGTTTTTG
shNLRP14	CCGGGCATCTGGATCTAGGATCAAACTCGAGTTTGATCCTAGATCCAGATGCTTTTTG
shNLRC3	CCGGCATCGCAGTGGCAGTGAGAGACTCGAGTCTCTCACTGCCACTGCGATGTTTTTG
shNLRC4	CCGGCCATACCTTCTATGATCTGTTCTCGAGAACAGATCATAGAAGGTATGGTTTTTG
shNLRC5	CCGGCCTGTAAGATTGACAACCAGACTCGAGTCTGGTTGTCAATCTTACAGGTTTTTG
shcaspase1 #1	CCGGCTACAACTCAATGCAATCTTTCTCGAGAAAGATTGCATTGAGTTGTAGTTTTTG
shcaspase1 #2	CCGGCACACGTCTTGCTCTCATTATCTCGAGATAATGAGAGCAAGACGTGTGTTTTTG
shAIM2#1	CCGGGGAACAATTGTGAATGGTTTGCTCGAGCAAACCATTCACAATTGTTCCTTTTTG
shAIM2#2	CCGGCCCGAAGATCAACACGCTTCACTCGAGTGAAGCGTGTTGATCTTCGGGTTTTTG
shGSDMD#1	CCGGGTGTGTCAACCTGTCTATCAACTCGAGTTGATAGACAGGTTGACACACTTTTTG
shGSDMD#2	CCGGCAGCACCTCAATGAATGTGTACTCGAGTACACATTCATTGAGGTGCTGTTTTTG
shKAP1#1	CCGGACTGTTATACAGCAAGGGAATCTCGAGATTCCCTTGCTGTATAACAGTTTTTG
shKAP1#2	CCGGGAGATCCTCAAGAGGAACATTCTCGAGAATGTTCCTCTTGAGGATCTCTTTTTG
shBHRF1#1	CCGGTGGATGGTTGGATTCATCAACCTCGAGGTTGATGAATCCAACCATCCATTTTTG
shBHRF1#2	CCGGATGGTTGGATTCATCAACAGGCTCGAGCCTGTTGATGAATCCAACCATTTTTG
